# Mapping the travel patterns of people with malaria in Bangladesh

**DOI:** 10.1186/s12916-020-1512-5

**Published:** 2020-03-04

**Authors:** Ipsita Sinha, Abdullah Abu Sayeed, Didar Uddin, Amy Wesolowski, Sazid Ibna Zaman, M. Abul Faiz, Aniruddha Ghose, M. Ridwanur Rahman, Akramul Islam, Mohammad Jahirul Karim, Anjan Saha, M. Kamar Rezwan, Abul Khair Mohammad Shamsuzzaman, Sanya Tahmina Jhora, M. M. Aktaruzzaman, Hsiao-Han Chang, Olivo Miotto, Dominic Kwiatkowski, Arjen M. Dondorp, Nicholas P. J. Day, M. Amir Hossain, Caroline Buckee, Richard J. Maude

**Affiliations:** 1 Mahidol-Oxford Tropical Medicine Research Unit, Bangkok, Thailand; 2 Centre for Tropical Medicine and Global Health, Nuffield Department of Medicine, University of Oxford, Oxford, UK; 3 Chittagong Medical College and Hospital, Chittagong, Bangladesh; 4 John Hopkins Bloomberg School of Public Health, Baltimore, USA; 5 Harvard T.H. Chan School of Public Health, Harvard University, Boston, USA; 6 BRAC (Building Resources Across Communities), BRAC Centre, Mohakhali, Dhaka, Bangladesh; 7 Dev Care Foundation, Dhaka, Bangladesh; 8 Shaheed Suhrawardy Medical College, Dhaka, Bangladesh; 9 Communicable Disease Control, Directorate General of Health Services, Dhaka, Bangladesh; 10 Filariasis Elimination, STH Control, Dhaka, Bangladesh; 11 National Malaria Elimination Programme, Dhaka, Bangladesh; 12 Vector-Borne Disease Control, World Health Organization, Dhaka, Bangladesh; 13 Big Data Institute, University of Oxford, Oxford, UK; 14 Wellcome Sanger Institute, Hinxton, Cambridge, UK

**Keywords:** Malaria epidemiology, Bangladesh, Population movement

## Abstract

**Background:**

Spread of malaria and antimalarial resistance through human movement present major threats to current goals to eliminate the disease. Bordering the Greater Mekong Subregion, southeast Bangladesh is a potentially important route of spread to India and beyond, but information on travel patterns in this area are lacking.

**Methods:**

Using a standardised short survey tool, 2090 patients with malaria were interviewed at 57 study sites in 2015–2016 about their demographics and travel patterns in the preceding 2 months.

**Results:**

Most travel was in the south of the study region between Cox’s Bazar district (coastal region) to forested areas in Bandarban (31% by days and 45% by nights), forming a source-sink route. Less than 1% of travel reported was between the north and south forested areas of the study area.

Farmers (21%) and students (19%) were the top two occupations recorded, with 67 and 47% reporting travel to the forest respectively. Males aged 25–49 years accounted for 43% of cases visiting forests but only 24% of the study population. Children did not travel. Women, forest dwellers and farmers did not travel beyond union boundaries. Military personnel travelled the furthest especially to remote forested areas.

**Conclusions:**

The approach demonstrated here provides a framework for identifying key traveller groups and their origins and destinations of travel in combination with knowledge of local epidemiology to inform malaria control and elimination efforts. Working with the NMEP, the findings were used to derive a set of policy recommendations to guide targeting of interventions for elimination.

**Electronic supplementary material:**

The online version of this article (10.1186/s12916-020-1512-5) contains supplementary material, which is available to authorized users.

## Background

Malaria has undergone a 54% reduction in mortality in Southeast Asia from 2010 to 2017 [1]. A major contributor to this has been widespread availability of effective artemisinin combination therapies (ACTs) as first-line antimalarial treatment [2, 3]. Their ongoing efficacy is critical to malaria control and elimination efforts worldwide. The identification of extended parasite clearance times in patients with malaria treated with artemisinins in Cambodia [3, 4], and the subsequent discovery of parasite mutations associated with this phenotype [5, 6] confirmed the presence of resistance to artemisinins. This has since also been found in Lao PDR, Vietnam, Thailand and Myanmar [7–9]. More recently, treatment failure to ACTs was found in Cambodia with resistance to both artemisinins and ACT partner drugs [10]. This has led to the rapid mobilisation of researchers and public health agencies to find solutions to monitor and contain artemisinin and ACT resistance [11–14].

In the history of malaria control, the emergence of antimalarial resistance has occurred repeatedly in Southeast Asia, for reasons that remain obscure, and spread via human migration throughout the region and subsequently to Africa [15–17]. Understanding how human migration contributes to the spread of malaria and resistance in the region has been identified as one of the most critical goals of current research and public health efforts on malaria, especially in the context of planned elimination, increasing international travel, the opening up of Myanmar and increasing travel of refugee populations [12, 13]. Bangladesh is a high population density country, with endemic malaria [18] in regions bordering Myanmar and India. This is coupled with strong migratory ties to these countries and to Africa [19–23]. It is therefore an important country to monitor for spread of malaria and resistance through human mobility.

Around 90% of malaria in Bangladesh is due to *Plasmodium falciparum* and around 85% of cases occur in the malaria-endemic Southeast [18, 24, 25]. The area of highest malaria transmission is the inland Chittagong Hill Tracts (Khagrachhari, Bandarban and Rangamati Districts), which, despite being sparsely populated, has around 90% of the malaria cases in Bangladesh [26]. Cox’s Bazar, a coastal town adjacent to Bandarban in the south of Chittagong Division, is situated on a major highway, which travellers frequently pass through on their way north and inland. It is hypothesised that migration of individuals within Bangladesh between the highly populated coastal areas around Chittagong and the rural Hill Tracts for farming, plantation work and logging, represent a grave risk for the dissemination of malaria. These sites could represent potential sources of malaria spread and an important sink for potentially resistant parasites to spread into and become established, as has happened in Southeast Asia [27]. So far, malaria epidemiology in Bangladesh has primarily been described through national incidence data known to be an underestimate of the true burden [28]. Information on migration and travel patterns of infected individuals from Bangladesh has been sparse. Studies have looked at international travel and malaria in Bangladesh but only at the national level using reported numbers of imported cases [29] or using airline and shipping data for the general population [30], whereas much of the international travel in malaria-endemic areas occurs overland between neighbouring countries. Other studies have used census data on migration of the general population [31, 32]. These may not be representative of people with malaria or sufficiently detailed to guide planning of interventions. Other studies have looked at domestic travel and malaria in Bangladesh in subgroups in limited areas including one which asked about prior history of travel at the district level of patients with severe malaria who had been treated in the tertiary referral hospital in Chittagong [24]. Galagan [33] reported internal migration of jhum (shifting cultivation) workers within two unions in Bandarban district from a surveillance study in that area.

Methods have been developed for modelling the impact of human mobility on the spread of malaria using travel surveys and mobile phone data with several approaches to constructing a source-sink map of malaria spread [34, 35]. To date, these studies have largely concentrated on African children [35–41], with a paucity of studies in Asia [18]. The WHO surveillance strategy states countries close to elimination should monitor high-risk populations such as migrants using active case detection routinely [42]. Methods for travel data collection and analysis vary widely, and the results have not been used to directly inform planning of National Malaria Elimination Programme (NMEP, formally known as National Malaria Control Programme (NMCP) activities. The analytical methods developed to date combine mechanistic mathematical models of malaria transmission with spatial analyses and routine data sources such as the primary surveillance data collected by NMCPs [35, 39, 43]. With some modification, and with collection of appropriate travel data, they are potentially suitable for assessing the impact of mobility on the spread of malaria and antimalarial resistance in Asia to help guide elimination strategy planning by NMCPs.

An observational study was conducted with demographic and travel surveys collected from unselected people with malaria in Southeast Bangladesh. A modelling analysis combining *P. falciparum* parasite population genetic data from patients in this study and aggregated connectivity data from the travel survey analysis with mobile phone call detail records and malaria incidence rates has been reported by Chang et al. [44]. This study demonstrated the use of a genetic mixing index measure to quantify parasite importation and identify likely locations of transmission but did not describe in detail which people were travelling, why they were doing so and where different groups travelled to and from which is essential information for the National Malaria Elimination Programme of Bangladesh to plan a strategy to reduce malaria in these groups to avert its spread through human movement. This complementary analysis is described in the present manuscript. The aims were to determine where people travelled during the time they were likely to have been infected, to quantify and map these patterns, to identify which demographic and occupation groups travelled the most, and to identify demographic groups at increased risk of malaria. The method described by Chang et al. was also limited in its generalisability as it required contemporaneous travel survey, genetic and cell phone data which is not available in most countries. This separate manuscript therefore had an additional aim to develop methods for mapping sources and sinks of malaria using only travel surveys together with incidence data and other publicly available information on population and forest cover that could be applied elsewhere. This was used as evidence to derive a set of policy recommendations for the NMEP to target these groups with interventions for elimination.

## Methods and materials

### Field study

**Fig. 1:**
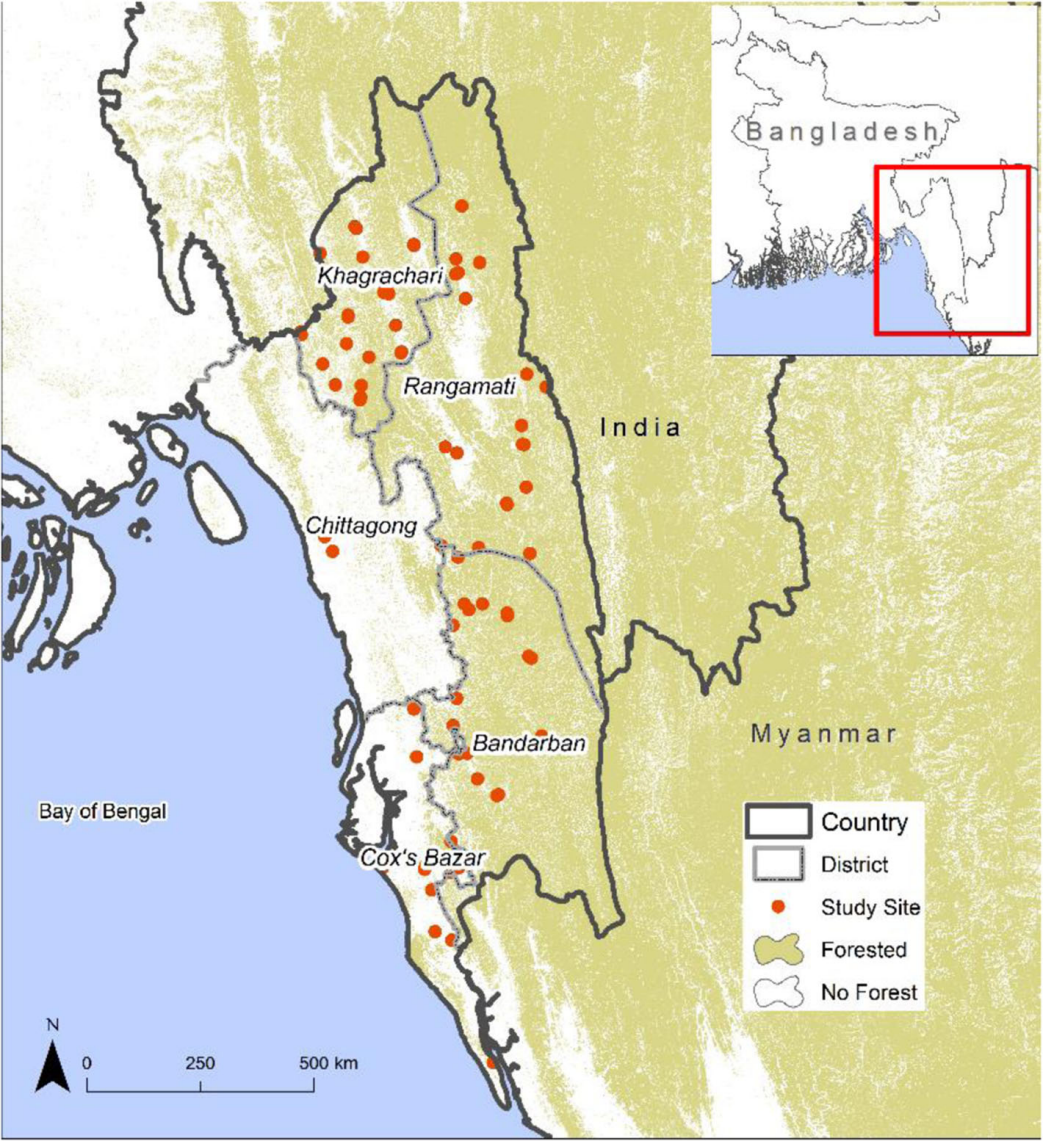
Study sites, districts and forest distribution

A prospective observational study was conducted with enrolment of patients at 57 study sites from January 1, 2015 to September 31, 2016, thus covering two malaria seasons (June to October). The study sites consisted of community clinics, health centres and hospitals located, as per Fig. 1, across 5 contiguous districts within Chittagong Division in Southeast Bangladesh, specifically Chittagong, Khagrachhari, Rangamati, Bandarban and Cox’s Bazar.

Ethical approval was obtained from the Oxford Tropical Research Ethical Committee, Bangladesh Medical Research Council Ethical Committee and Harvard University Human Research Protection Program.

Patients of all ages self-presenting to the study sites and having a positive test for malaria were recruited provided written consent was obtained. A positive test was the presence of asexual stage parasites on microscopic examination of a smear of peripheral blood including individuals with one or more species of Plasmodium on microscopy or rapid diagnostic test [26]. RDTs were provided by BRAC (formerly Bangladesh Rural Advancement Committee, presently Building Resources Across Communities) and the NMEP [26]. There was a change in RDT brand supplied from the First Reponse Malaria Ag (pLDH/HRP2) Combo Card test (Premier Medical Corporation Ltd., Gujarat, India) to One Step test for Malaria Pf/Pv Ag MERISCREEN (Meril Diagnostics Pvt. Ltd., Gujarat, India) in the early part of the study period in mid-2015 when recruitment had just commenced (Additional file 1: Table S1). Both were able to detect *P. falciparum*, non-*P. falciparum* and mixed species. Upon enrolment, an additional blood sample was taken using a finger prick onto filter paper for parasite DNA analysis.

Recruited patients were administered a two-page survey consisting of demographic information (age, gender, place of residence, occupation and place of work, including average number of days in a week spent travelling to and from work), any other travel in the preceding 2 months other than work which included specific questions on nights away, travel to forest, frequent travel other than work, infrequent travel and travel to other countries. This survey was developed and optimised prior to the study through discussion with the NMEP and local investigators followed by piloting in the field. It was designed to collect information wanted by the NMEP to help them plan interventions for these groups. Information on travel is collected routinely by many National Malaria Control Programmes around the world, as part of case investigations, as recommended by the WHO [42]. The survey used in this study was more detailed than the routine travel information and was developed with piloting alongside detailed travel diaries in villages in Cambodia and Bangladesh, and improved iteratively based on user feedback and comparison of results. Two months was chosen as the time period during which malaria transmission was most likely to occur, and also over which recall was found to be reliable. This paper presents a detailed descriptive analysis of the travel survey data. A subset of the data fields was used for quantifying travel and for modelling to estimate parasite importation as described earlier [44]. The present manuscript describes the full range of data collected including detailed demographics and different types and motivations for travel.

### Secondary data

Administrative division (admin) boundary shapefiles for divisions (admin 1), districts (admin 2), upazilas (subdistrict/admin 3) and unions (admin 4) were obtained from the Government of Bangladesh. The study area in Chittagong Division comprised 5 districts, 58 subdistricts and 384 unions. It also included patients whose residence was outside the study area but travelled to a health facility within the study area for a diagnosis. Complete and accurate information on the locations of villages in the study area were not available, so the analysis was limited to unions as the smallest spatial unit.

Data on numbers of confirmed malaria cases in the whole country at the subdistrict (upazila) level during the period of the study were obtained from the NMEP [45]. Incidence data was used, as prevalence data had only been collected in standalone surveys from a limited number of sites [28, 46]. Data on population by administrative subunit from the 2011 census were provided by the Bangladesh Bureau of Statistics and available up to the subdistrict (upazila) level.

Annual forest cover (percentage of pixel, 1 pixel = 30 m × 30 m at the equator) was calculated from raster files downloaded from Hansen et al. (https://earthenginepartners.appspot.com/science-2013-global-forest) [47] using the “Zonal statistics” package in ArcGIS. When comparing forest cover by union from satellite remote sensing data to survey results, a value of percent forest cover for the union was assigned to each person (“per person forest cover”).

### Analysis

The results from the travel survey were combined with incidence data from the National Malaria Elimination Programme (NMEP), census data and forest cover from satellites, to identify key demographic groups of travellers and map sources and sinks of spread and routes of travel. For data management and statistical analysis, R version 3.3 was used. For spatial analysis, ArcGIS version 10.3 (ESRI, Redlands, CA, USA) was used. Annual parasite index was calculated as the number of confirmed cases in an area in 1 year/population in that area in the 2011 census × 1000. Population growth rate estimates were not available at the subdistrict level, so a fixed population size was used throughout.

An exploratory analysis was performed combining demographic and travel data to identify which groups were travelling. Data on study subjects was compared to data, by gender and 5 yearly age bands, from the census covering the study area to identify groups at higher risk of malaria. Travel matrix distances were calculated from centroid longitude and latitude of each Union polygon using the “Geosphere package” in R [48]. This was validated using packages “XY to line” and “Near Neighbour” in ArcGIS. Actual coordinates of study sites were used when measuring distance travelled from the centroid of a residence union to the study site.

Unlike the previous analysis which focused on number of trips [44], the amount of travel was measured as (1) number of people travelling from residence, (2) total number of days away from residence and (3) nights away from residence to a particular destination over 2 months. Travel from residence to study site was also included.

A multiple correspondence analysis was utilised to determine the key demographic factors contributing to the most amount of variance in the extent of travel, using the R package “Facto Mine” [49]. Multivariable logistic regression was then performed to determine the odds ratios for demographics to better understand the likelihood of certain demographic groups travelling.

Finally, a simple empirical approach was taken to examine what the possible sources and sinks of malaria spread were at the subdistrict level. Conceptually, a source was a subdistrict from which malaria was likely to be exported to other subdistricts. A sink was a subdistrict to which malaria was likely to be imported from other subdistricts. This definition of net exporter, i.e. source and net importer, i.e. a sink, is well established in the ecological literature [50]. In addition to travel, whether a subdistrict is a source, sink or both can be influenced by relative transmission intensity (malaria more likely to be exported from areas of high transmission to low transmission than vice versa [34]). It could also be biased by different enrolment rates in different locations, thus requiring some form of adjustment/normalisation for this.

There is no single agreed methodology for identification of sources and sinks, using measured travel patterns of malaria patients. Recent studies inferring malaria sources and sinks in Madagascar and Namibia used call data records and malaria prevalence maps [34, 35]. In this paper, two different methods were used, and results compared. The main difference between the two different methods was the incorporation of more accurate estimates of API in method 2, with method 1 being more suitable for areas where API is less precisely known or is unavailable. The origin was assumed to be the subdistrict of residence. Subdistricts were only considered to be sources or sinks if both origin and destination had API > 1, i.e. a moderate risk of malaria transmission as defined by WHO [13]. This was chosen as a conservative arbitrary cut-off to exclude stochastic results from areas with very low burden or low risk which could lead to inappropriate allocation of resources by the NMEP to target these low-risk areas (API < 1). Source and sink maps for malaria were produced using the following equations for each of number of enrolled cases, number of days away from residence and number of overnight stays away from residence over the preceding 2 months.

Given an origin-destination matrix (*X* by *Y*) with *X* origins (residences) and *Y* destinations where:

*M*_o_ = amount of travel from a given origin subdistrict to other endemic destination subdistricts

*M*_d_ = amount of travel to a given destination subdistrict from other origin subdistricts

API_o_ = API of origin subdistrict

API_d_ = API of destination subdistrict

*r* = number of enrolled cases resident in origin subdistrict

Method 1: Weighting by *r*

1. $$ \mathrm{Source}=\mathrm{Rank}\left(\sum \limits_{1\kern0.5em }^X{M}_{\mathrm{d}}/r\right) $$
2. $$ \mathrm{Sink}=\mathrm{Rank}\left(\sum \limits_{1\kern0.5em }^Y{M}_{\mathrm{o}}/r\right) $$

Method 2: Weighting by *r* and API

3) $$ \mathrm{Source}=\mathrm{Rank}\left(\sum \limits_{1\kern0.5em }^X{M}_{\mathrm{d}}{\mathrm{API}}_{\mathrm{o}}/{\mathrm{API}}_{\mathrm{d}}r\right) $$
4) $$ \mathrm{Sink}=\mathrm{Rank}\left(\sum \limits_{1\kern0.5em }^Y{M}_{\mathrm{o}}{\mathrm{API}}_{\mathrm{o}}/{\mathrm{API}}_{\mathrm{d}}r\right) $$

Sources with fewer than 5 enrolled cases and sinks with fewer than 5 incoming cases were removed because of high uncertainty due to small numbers, again to avoid stochasticity. The sources and sinks were then ranked and grouped in quantile bands. Method 1 is suitable for areas where only the range of API ≤ 1 or > 1 or no API is known. Method 2 assumes source and sink can be influenced by relative transmission and requires that API or prevalence data is available for the origin and destination [34, 44].

## Results

In total, 2100 unselected patients were recruited from June 2015 to September 2016. Enrolment in Rangamati district commenced from April 2016 onwards. Ten patients were excluded as they did not have confirmed malaria leaving 2090 enrolled patients with malaria. Enrolment broadly followed the pattern of seasonal peaks in incidence reported to NMEP with a slower uptake at the beginning of the study period (Additional file 1: Figure S1).

### Demographics

Males comprised 67% of the study population compared to 51% in the 2011 census for the 5 study districts in Chittagong Division (Additional file 1: Figure S3). The proportion who were male aged between 15 and 39 years was higher at 72% compared to 49% in the census (*P* < 0.001). When correcting for multiple comparisons across 5-year age bands, there was a higher proportion of males in age bands from 15 to 39 years in the study population than in the census (Bonferroni method, 17 age bands, significance *p* < 0.003). Children under 15 years comprised 34% of the study population, of which 55% were male. There were no differences in the proportion of adult females or children of either gender in the study population compared to the census. There were also no substantial differences in age or gender distribution in the study population compared to the NMEP surveillance data from the whole of Chittagong Division during the study period.

The five most reported occupations were farmer (21%), student (19%), forestry (16%), child (16%) and housewife (11%). Full details of occupations reported are shown in Additional file 1: Table S4. The majority of farmers (72%) were engaged in paddy farming. Of the people who worked in the forest, 21% worked in jhum cultivation (slash and burn farming), especially in the hilly areas, and another 21% worked in plantations close to the forest. The top three reported occupations for males were farming (28%), forest-related (21%), and student (16%) and for females, housewife (32%), student (24%) and child (22%). Of the 16 cases who reported their residence as outside the study catchment area, 14 were in the military.

#### Plasmodium species

Routine diagnostic testing found *Plasmodium falciparum*, *P. vivax* and mixed infection in 74% (*N* = 1543), 16% (*N* = 332) and 10% (*N* = 215) of enrolled individuals compared to 64%, 11% and 25% in the NMEP surveillance data for Chittagong Division during the same period. Only the proportion with mixed species infection was higher in the surveillance data (*p* < 0.001). Details of district, age, gender, occupation and forest status distribution by species are shown in Additional file 1: Tables S2-S5 and Figure S3. The proportion of children under 15 years of age was higher for *P. vivax* (45%) compared to *P. falciparum* (34%) (*P* < 0.001). No other significant differences were noted.

#### Spatial distribution of cases

**Fig. 2:**
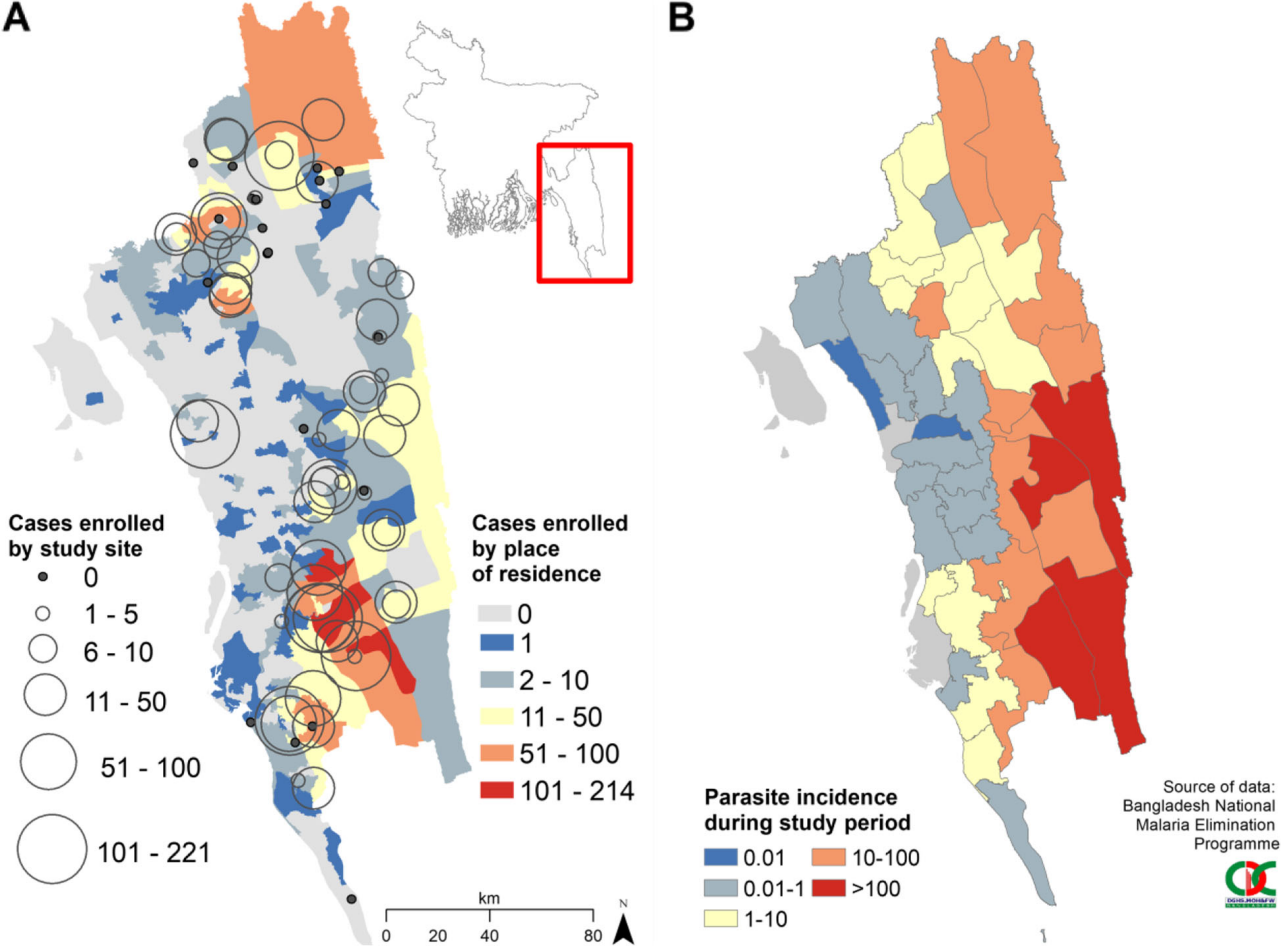
Spatial distribution of **a** total malaria cases enrolled in the study by study site and place of residence and **b** malaria incidence reported by NMEP during the study period

Bandarban district in the south of Chittagong Division contributed 1033 (49%) of the enrolled cases (Fig. 2a) followed by Khagrachhari district in the north with 416 (20%) cases. The geographic distribution of enrolled cases broadly followed the spatial distribution of total cases reported to the NMEP (Fig. 2b). There was under-recruitment in some of the remote forested border areas in Bandarban (Additional file 1: Figure S2). Lama in Bandarban was the subdistrict with the highest recruitment of cases with 574 (27%) followed by Ramu with 254 (12%). There were no cases resident in the city of Chittagong, with Chittagong district having cases mainly in the forest fringe areas, such as Fatikchhari (11 cases), Lohagara (8 cases) and Banshkhali (6 cases) subdistricts.

### Overall travel

Of the 2090 enrolled patients, 1631 (78%) reported travel within the previous 2 months. Of the patients who travelled, 729 (45%) reported travelling only for work, 178 (11%) reported travel for purposes other than work during the day and 724 (44%) reported overnight stays.

#### Travel from residence to the study site

**Fig. 3:**
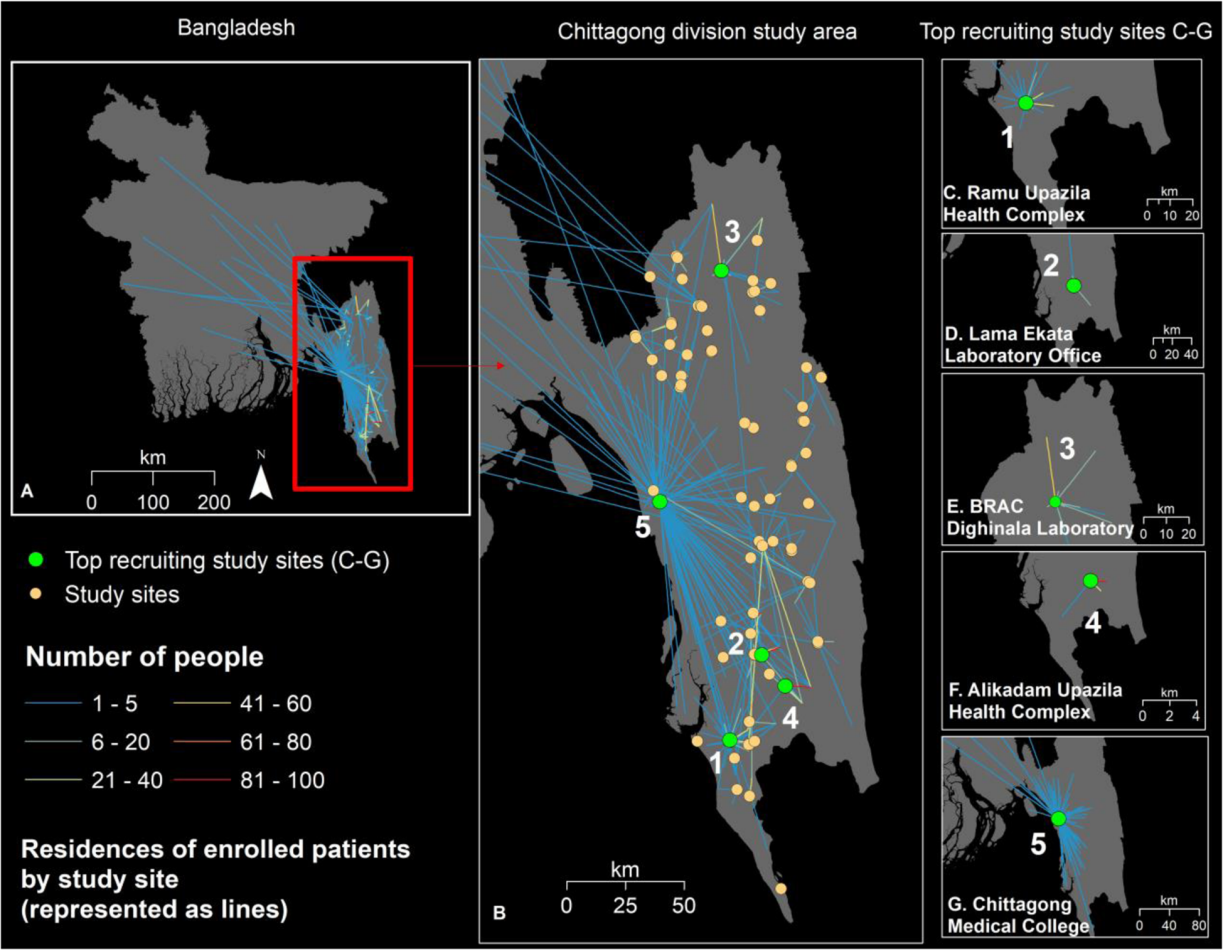
Number of people travelling on a given route from place of residence to study site or health facility. **a** Whole country and **b** Chittagong Division (highlighted in green are the locations of the 5 study sites (1–5) with highest enrolment). The panels on the right show in descending order the health catchment by residence of patients at the 5 study sites (1–5) with the highest enrolment. **c** Ramu Upazila Health Complex 221 (10%), **d** Lama Ekata Laboratory Office 195 (9%), **e** BRAC Dighinala Laboratory 163 (8%), **f** Alikadam Upazila Health Complex 120 (6%) and **g** Chittagong Medical College Hospital 115 (5%)

The overall geographic pattern of travel from residence at the union level to the study site is shown in Fig. 3. It can be seen in 3a and 3b that these patterns are a combination of health facility catchment areas for people living locally plus some people travelling long distances across the country from their residence before presenting to a health facility at their destination. Panels c–g show the top 5 study sites which recruited the most cases (39%) in descending order of enrolled cases: (1) Ramu Upazila Health Complex, (2) Lama Ekata Laboratory Office, (3) BRAC Dighinala Laboratory, (4) Alikadam Upazila Health Complex, (5) Chittagong Medical College Hospital (CMCH). Ten percent of all cases from the study were enrolled at Ramu Upazila Health Complex. This is located in Cox’s Bazar, a coastal district in the south of Chittagong Division, itself an area with relatively low incidence but situated adjacent to the high-incidence areas in Bandarban district. There was a separate ongoing malaria research project at this site during the study period which may have accounted for the high enrolment. CMCH, the main tertiary referral hospital in Chittagong city (otherwise referred to as Chittagong city corporation), had the widest catchment area with zero cases resident in the city (where there is thought to be no malaria transmission), 51% resident in other subdistricts in Chittagong district, 47% resident in the Chittagong Hill Tracts and 2% resident in another division. The sites in Lama, Dighinala and Alikadam had smaller catchment areas and relatively high malaria incidence rates.

#### Travel from residence to reported destination

Looking at the distribution of travel from residence to reported destination by administrative unit, of the 1631 people who reported travel, 1064 (65%) travelled to another location only within the smallest spatial unit of analysis, the union and 261 (16%) travelled outside the union but only within their own district. For longer distance travel, 273 (17%) cases travelled to another district within Chittagong Division, 23 (1%) to another division in Bangladesh outside the study area and 14 (1%) to another country. International travel comprised 6 people visiting Myanmar from Bandarban district, 4 going to India from Rangamati district, 1 to the Kingdom of Saudi Arabia for the Haj, 1 to the Democratic Republic of the Congo for peacekeeping, and 1 to Mozambique for business. One person cited residence in Lunglei, India, and visited Rangamati district for treatment.

Most travel outside unions of residence was in the south of the study region between Cox’s Bazar district (coastal region) to forested areas in Bandarban (31% of days and 45% of nights of overall travel, rising to 64% and 72% respectively for inter-district travel). Accounting for total travel reported by residents in Cox’s Bazar district both within and outside Cox’s Bazar, travel to Bandarban amounted to 32% by day and 80% by night (Additional file 1: Figures S5A and S5B). Travel was clustered in the north and south of the Chittagong Hill Tracts, with less than 1% of travel days and nights between northern and southern districts (Additional file 1: Figures S5 and S6). Most of the inter-district travel from Chittagong district, which is the site of the division capital and an area of low malaria endemicity, was to the malaria-endemic district of Bandarban (95% of travel days and 83% of overnight stays).

**Fig. 4:**
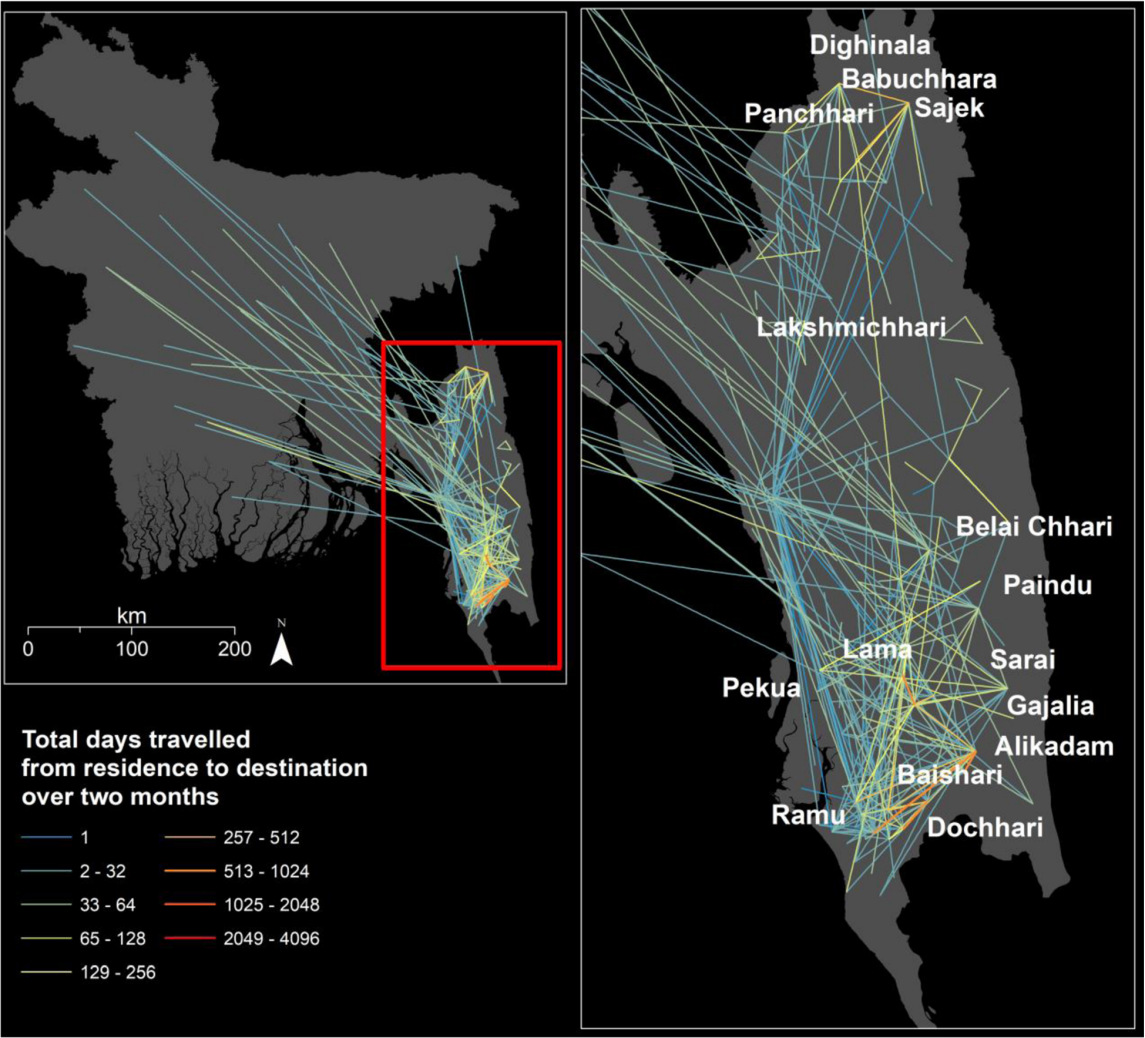
Days travelled from residence to destination over 2 months

The overall geographic pattern of total days away from place of residence over the previous 2 months at a reported destination is shown at the union level in Fig. 4. A total of 87,683 days of travel were reported by all cases over the 2 months. In total, 80% of travel days were within the same union (Additional file 1: Table S6). Of days travelled, 81% were for work, of which 87% were within the same union. 12% of travel days were to a different district from their district of residence. For people travelling to the forest, travel outside the district of residence comprised 35% of travel days. Lama union had the most travel days within the union with 6213 (7%) days, followed by Alikadam union at 5319 days (6%). Out of the top 5 within union travel destinations, 3 were within Lama subdistrict (Additional file 1: Table S8). Looking at travel outside the union, the most travelled route was between Kachhapia in Ramu, Cox’s Bazar district to a neighbouring union Docchari in Bandarban district at 2006 days (2%).

The overall geographic pattern of travel by nights spent away from home (Additional file 1: Figure S4) was similar to that for days. In total, 10,817 nights were spent away from home with Alikadam union in Bandarban district reporting the most amount of overnight travel (Additional file 1: Table S9), all within the same union (593 or 5%). The highest recorded travel by nights between unions was from Kachhapia union, in Ramu subdistrict (Cox’s Bazar district) to Docchari union in Naikhongchhari subdistrict (Bandarban district) at 544 nights (5%). Only 34% of cases travelled within the same union when spending nights away from home compared to 48% travelling for non-work purposes during the day (Additional file 1: Table S7). The majority of nights (81%) spent away were for travel to the forest. Of cases reporting overnight travel, 44% travelled outside their district of residence. Over half the travel from outside the study area (134 nights, 55%) was to Bandarban district.

Residents from Chittagong district tended to travel further than other residents in the study area (median (IQR) 39 (22–56) vs 18 (10–32), *p* = 0.0004), as did patients from outside the study area (*p* < 0.001), Additional file 1: Figure S9.

#### Malaria sources and sinks

##### Sources

Lama and Alikadam subdistricts in Bandarban district, Chakaria and Ramu in Cox’s Bazar and Dighinala in Khagrachhari had the highest numbers of travellers to other endemic subdistricts (Additional file 1: Figure S10A). Using method 1, adjusting for the number of enrolled cases by subdistrict of residence (Additional file 1: Figure S10B), the top 4 ranked source subdistricts were all in Cox Bazar’s district (Pekua, Chakaria, Ramu and Ukhia—forming a contiguous region), with travel mostly to Alikadam, and Naikhongchhari subdistricts in Bandarban district for farming and forestry work. The next highest ranked sources were Kaptai (but with only 3 outgoing visitors and 6 enrolled cases) in Rangamati, adjoining a large lake, followed by Panchhari and Dighinala in Khagrachhari district with most travel to Sajek union in Baghaichhari. Using method 2, adjusting for both enrolment and origin and destination malaria API, the top 6 ranked sources were Belaichhari in Khagrachhari district, Alikadam in Bandarban district, Lama in Bandarban district, Ramu in Cox’s Bazar district, Matiranga in Khagrachhari district and Chakaria in Cox’s Bazar district (Additional file 1: Figure S10C). Including API had a major influence on the results. For example, Belaichhari had the second highest API in the Chittagong Hill Tracts but only 10% of cases travelled out from this subdistrict. Being next to the Indian border, the main means of travel in this hilly area was through a waterway and people travelling out of this subdistrict were likely to stay overnight. Lama and Alikadam were higher ranked as sources using method 2, with higher API, and substantial numbers of travellers to nearby subdistricts, in contrast to ranked sources adjusted solely for enrolment.

##### Sinks

Subdistricts with the highest numbers of incoming visitors from endemic areas were as follows: Naikhongchhari in Cox’s Bazar district and Alikadam in Bandarban district with visitors from Ramu, followed by Baghaichhari in Khagrachhari district, Bandarban Sadar, Lama in Bandarban district, Cox’s Bazar Sadar in Cox’s Bazar district and Thanchi (remote forested area) in Bandarban district (Additional file 1: Figure S10D). The top ranking sinks, when adjusting for enrolled cases at origin (method 1), were Bandarban Sadar, Khagrachhari Sadar, Alikadam, Thanchi, Dighinala and Rowangchhari (another remote forested area in Bandarban district) (Additional file 1: Figure S10E). Method 2 gave similar results, but subdistricts with lower APIs such as Cox’s Bazar Sadar, Chakaria, Lohagara (forest fringe area near Chittagong) and Khagrachhari Sadar were ranked higher than Bandarban Sadar. Thanchi and Alikadam had the highest change in ranking from top 25% to the bottom 25% due to their higher API. Similar results were noted when considering days travelled and overnight stays (results are included in Additional file 1: Figure S11 for sources and S12 for sinks), with higher numbers of travellers and longer travel duration moving an area up the ranking.

### Travel and demographics

#### Age and gender

**Fig. 5:**
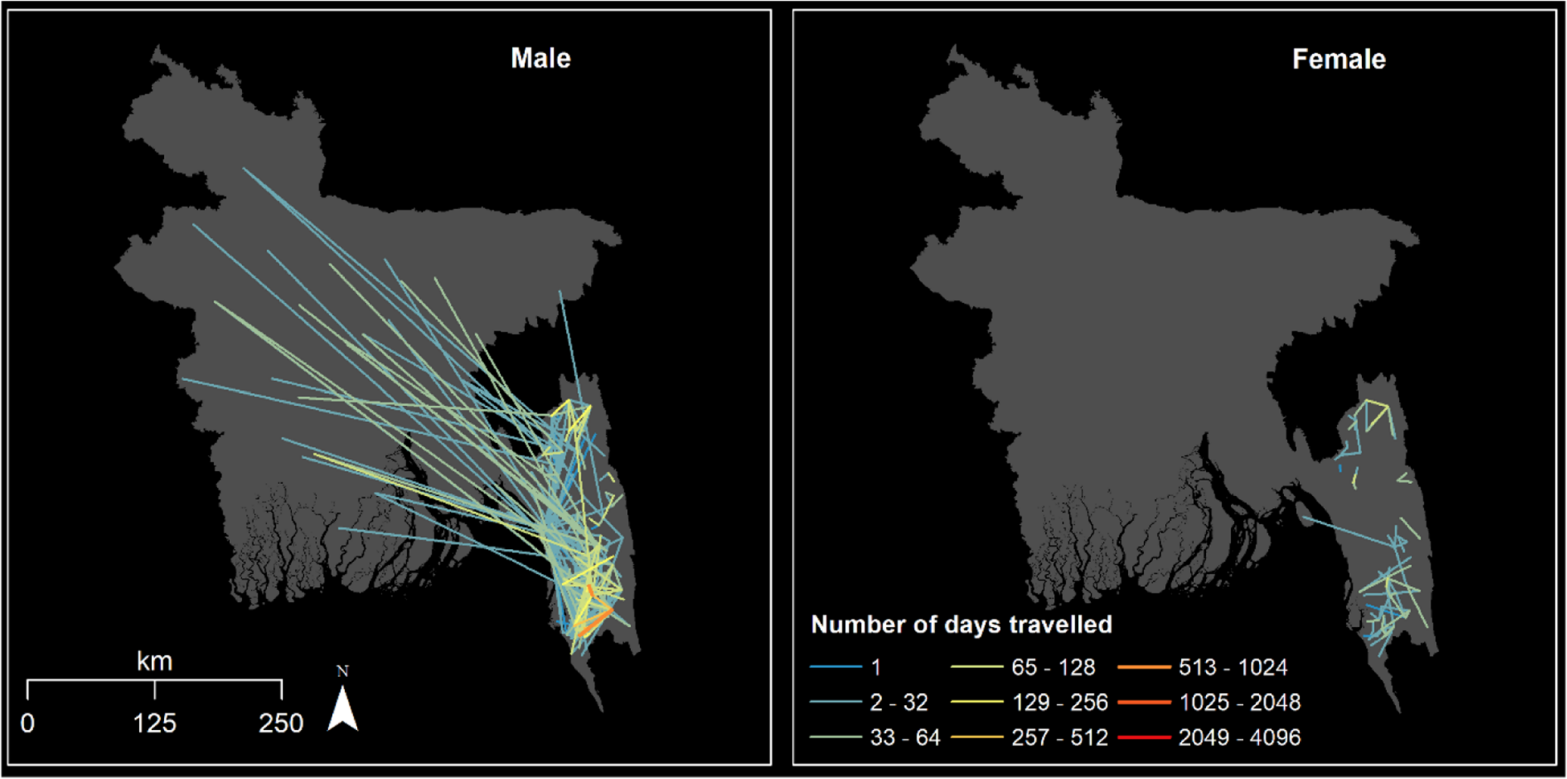
Numbers of days of travel between residence and destination over 2 months by gender

Travel patterns varied substantially between different demographic groups. For instance, men travelled further and more compared to women (Fig. 5, Additional file 1: Figure S13, and Table S12). A total of 100% of men aged 15–39 years travelled compared to 65% for the rest of the study population. In total, 37% of men aged 15–39 travelled outside their subdistrict of residence compared to 15% for all other men (*p* < 0.001).

#### Occupation

The geographic pattern of days of travel also differed greatly by occupation (Additional file 1: Figure S14). Farmers travelled the most (27,409 or 31% of days travelled). Children were reported to be either a student or a child when asked about occupation. Of the those who reported travel, only 14 children (28%), and 87 students (22% of the subgroup) travelled outside their union of residence. The distance travelled by military was the furthest, with 23% of their reported travel days greater than 100 km. Those with forestry-related occupations contributed the most amount of travel when looking at nights spent away from residence (3806 nights or 35%), forming two clusters of travel in the north and south of the Hill Tracts.

#### Odds of demographic factors and travel to another area

A univariate analysis was done by whether a group travelled or not, and the extent of their travel, i.e. outside the union, subdistrict or district for the following demographic factors—age, occupation, travel to forest and gender. Women, housewives, children aged under 5 years, people who lived in the forest and who did not visit the forest were less likely to travel (see Additional file 1: Figure S16 for results).

Significant factors from the univariate analysis were then included in a multivariate logistic regression to determine which groups travelled the most outside the administrative boundary of their place of residence (Table 1). The following groups were not included due to possible confounding: occupation listed as child (defined as age < 15 years), and housewife. Forest visits were not included as a separate factor due to overlap with forest-going occupations.

**Table 1:** Odds ratios of demographics in relation to travel from multivariate analysis

Predictors	Outside union			Outside upazila			Outside district		
	Odds ratios	CI	*p*	Odds ratios	CI	*p*	Odds ratios	CI	*p*
Age 0–4	0.17	0.07–0.43	**< 0.001**	0.22	0.08–0.63	**0.005**	0.43	0.12–1.57	0.202
Age 5–14	0.50	0.26–0.93	**0.030**	0.57	0.28–1.17	0.123	0.93	0.38–2.25	0.867
Age 15–24	1.58	0.93–2.71	0.092	1.64	0.90–2.98	0.104	2.38	1.13–4.98	**0.022**
Age 25–49	1.91	1.13–3.24	**0.016**	2.03	1.13–3.65	**0.018**	3.05	1.47–6.32	**0.003**
Male	2.51	1.87–3.37	**< 0.001**	3.64	2.49–5.33	**< 0.001**	4.10	2.55–6.60	**< 0.001**
Forest dweller	0.56	0.43–0.73	**< 0.001**	0.59	0.43–0.80	**0.001**	0.51	0.35–0.73	**< 0.001**
Student	2.57	1.64–4.02	**< 0.001**	1.43	0.82–2.47	0.204	1.52	0.76–3.03	0.235
Farming	1.18	0.79–1.74	0.419	1.20	0.76–1.90	0.441	1.86	1.05–3.29	**0.034**
Forestry	1.81	1.21–2.70	**0.004**	1.69	1.05–2.70	**0.030**	2.28	1.27–4.08	**0.006**
Labourer	2.71	1.53–4.79	**0.001**	2.56	1.38–4.74	**0.003**	3.37	1.66–6.84	**0.001**
Other	2.04	1.16–3.60	**0.014**	2.29	1.23–4.28	**0.009**	3.43	1.66–7.07	**0.001**
Business	2.12	1.14–3.97	**0.018**	2.32	1.18–4.54	**0.014**	3.37	1.58–7.22	**0.002**
Military	2.03	1.08–3.79	**0.027**	2.72	1.40–5.32	**0.003**	4.56	2.16–9.62	**< 0.001**
Observations	2090			2090			2090		

Bold values denote statistical significance at the *p* < 0.05 level

It was found that children under 15 years of age and women were least likely to travel beyond the union of residence (Table 1). Children and women also comprised the largest group amongst forest dwellers (63%). Students were significant for travel only beyond the union, but not further. Of those who reported travel, 44% of students aged 15–19 reported travel beyond the union, compared to 20% beyond the subdistrict. Males aged 25–49 were significant for travel at all administrative levels and consisted mainly of forest workers, farmers who reported travel to forest and labourers. Two thirds (63%) of labourers travelled to the forest from Cox’s Bazar to Bandarban. Military and businessmen travelled the furthest (*p* < 0.001).

This analysis was then restricted to subsets of travellers from the top quartile of predicted source subdistricts who travelled to another subdistrict derived using method 1 (Additional file 1: Table S13). The majority of the enrolled cases in source subdistricts were resident in Cox’s Bazar district (295 or 14% of total enrolled), followed by Khagrachhari district (118 or 8%). The odds ratios were different from the overall pattern when considering the whole study region. Military were less likely to travel if from a source subdistrict than from a non-source subdistrict, i.e. for cases resident in Cox’s Bazar or Khagrachhari district more than 90% travelled within the same district compared to cases originating elsewhere (Additional file 1: Figure S17A). Similarly, for forestry and farming occupations, of those residing in Cox’s Bazar district, 57% and 80% travelled to neighbouring subdistricts in a different district (i.e. Bandarban) rather than within Cox’s Bazar district. Heatmaps of demographic factors illustrating the proportions of travel between different districts are provided in the Additional file 1: Figure S17: A-I.

### Travel to forest

Patients were asked about travel to the forest and the reason for this travel. A third of cases reported that they live in the forest (“dweller”, 31%), a third reported having visited the forest (“visitor”, 32%) and just over a third (37%) reported not staying in or visiting the forest (“no visits”). There were no differences in forest residence or visit status between those with different malaria species (*P. falciparum* vs. *P. vivax* vs mixed).

#### Forest travel and demographics

Forest dwellers had proportionally more children, 353 (54%) and women, 299 (46%) compared to the groups who did not visit the forest, 346 children (45%) and 294 women (38%), and also for those who reported forest travel, 62 children (9%) and 106 women (16%) (Additional file 1: Table S15).

When looking at reported forest travel (“forest visitor”) by age (5-year bands) and gender (Additional file 1: Figure S18), males aged 25–49 years were over-represented, 303 (54%) compared to the group not visiting the forest (“No forest visits”) (137 (29%), *p* < 0.0001). This group was also over-represented compared to the study area census population, where males aged 25–49 constituted 32% of the census population (*p* < 0.001). Similarly, young males age 0–14 were under-represented, 27 (5%) in those who visited the forest compared to those who did not (184 (39%), *p* < 0.001). These differences were also found when correcting for multiple comparisons, as described earlier. There were no significant differences in the age or gender of the “forest dweller” or “no forest visit” groups compared to the census data for those living in Chittagong Division. Forest dwellers travelled shorter distances overall (11 km, 7–23), compared to both forest visitors (21 km, 11–38, *p* < 0.001) and “no forest visit” group (23 km, 11–39, *p* < 0.001). There was no significant difference in overall distances travelled between forest visitors and the no forest visit group (*p* = 0.4939). The forest visit group travelled more by days and nights compared to the no forest visit group (*p* < 0.001) (Additional file 1: Table S14, Figures S19, and S20).

#### Reasons for forest travel

**Fig. 6:**
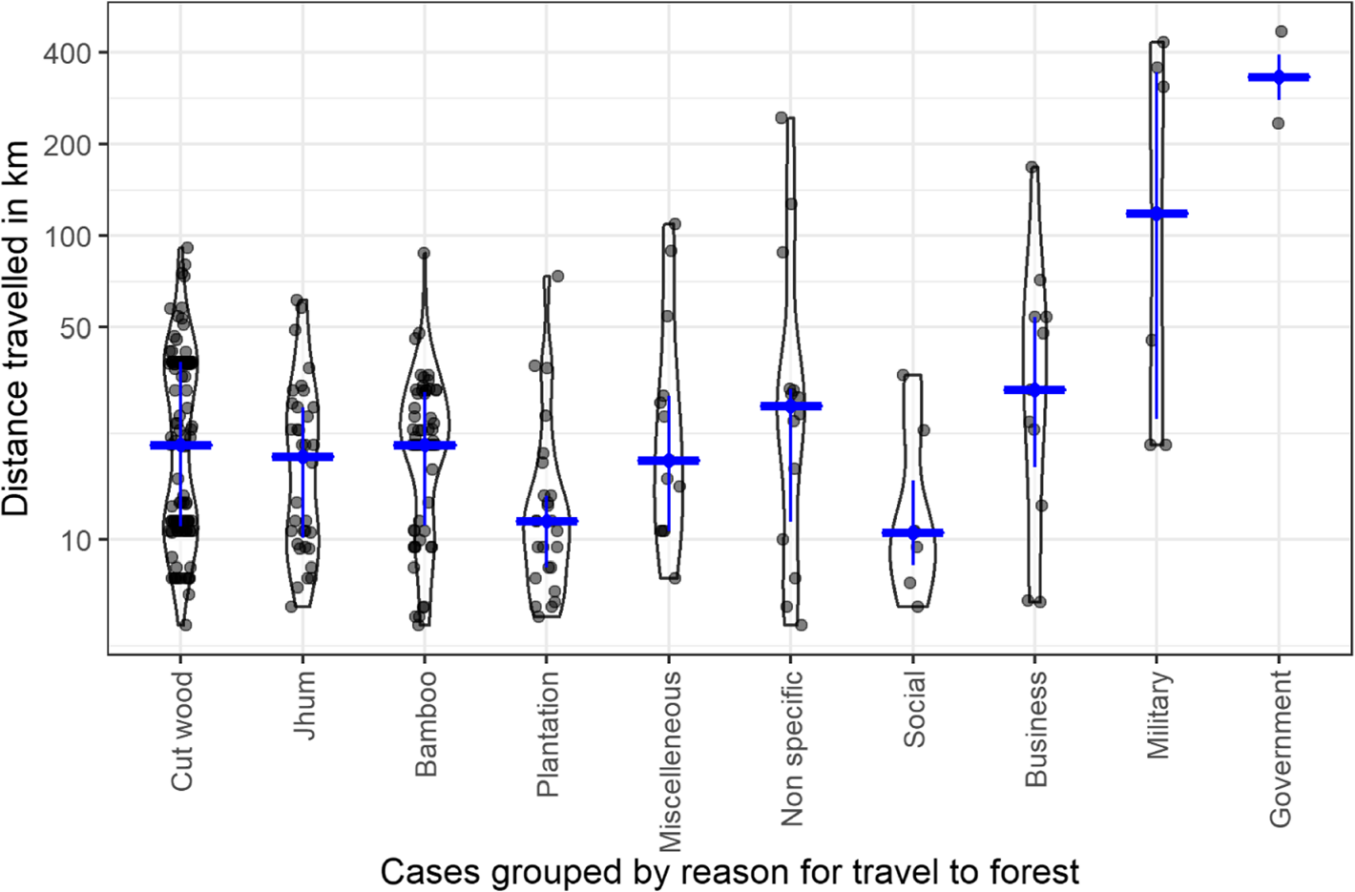
Distance travelled (km) by reasons for visiting forest

Figure 6 shows reported reasons for travel to forest. For jhum farming, people reported various reasons such as farming paddy, vegetables and turmeric. A quarter of the people travelling to plantations specified working in a rubber plantation (17 cases or 23%). Only 1 person reported hunting as a reason for visiting the forest. The median distance travelled to the forest was 21 km (IQR 11–34). Again, people who travelled for military (27 km, IQR 20–177) and government reasons travelled the furthest (293 km, IQR 247–352)

Study subjects were also asked about reasons for non-work travel which were reported by 316 cases. Of this subset, 37% reported travel for social reasons, 28% reported going to the market and a further 8% reported travel for forest-related activities. The median distance travelled for non-work travel was 17 km (IQR 8–33 km). Additional file 1: Figure S22 summarises reasons for non-work travel with distances travelled. People travelled the furthest for forest activities (reported as part of frequent non-work activity) with a median distance of 61 km and the shortest distances when going to the market at 7 km median distance. See Additional file 1 for a detailed breakdown of reasons for travel, including the “miscellaneous” group.

#### Forest travel and forest cover

Of the group who worked in the forest (as part of their occupation), 12% did not report visiting the forest in the preceding 2 months. Informal feedback from study staff revealed that reported forest travel in the study may have been affected by subjectivity regarding the definition of forest, in that different people had different ideas about what is meant by forest.

The median (range) forest cover by union for unions of residence in the study area was 24% and in the 3 Chittagong Hill Tracts (CHT) districts 67.5%. The overall per-person forest cover was 68% in the study area. There was no significant difference (Additional file 1: Figure S23A and B) in per-person % forest cover between unions of residence of people who reported living in the forest (median 67% forest cover, *n* = 657) and those who did not (median 68% forest cover, *n* = 1433). Unions of residence for enrolled patients in Cox’s Bazar and Chittagong districts had median forest cover of 4.5% and 9% respectively, and a per-person forest cover of 23 and 11% respectively. The 3 Chittagong Hill Tract districts had a median per-person forest cover of 68%. The border areas near India and Myanmar were heavily forested with 75% to 100% cover Additional file 1: Figure S23A. Looking at the travel destination of people who reported having travelled to the forest, especially in Bandarban district, they mostly visited heavily forested regions near the border (56% visited unions with ≥ 75% forest cover), compared to people who did not report visiting the forest (39%, *p* = < 0.001, Additional file 1: Figure S23B). Forest cover in destination unions for people who reported visiting the forest was 77%, higher than in destination unions for those who did not report visiting the forest (67%, *p* value < 0.001). The highly forested areas in the study area also had higher malaria incidence rates (median API 50.4 vs 2.7, *p* < 0.001), and lower population density (median 58 people per square km vs 692 people per square km, *p* < 0.001, Additional file 1: Figure S24).

## Discussion

Bangladesh has a goal to eliminate malaria by 2030 and is currently following the national strategic plan 2017–2021 [45], as per the WHO technical guidance [51]. This includes interrupting local transmission, preventing re-establishment and preventing emergence of *P. falciparum* strains resistant to artemisinins. Understanding and quantifying patterns of travel by malaria patients, sources of malaria spread and importation risk are important to guide strategies and interventions to achieve and sustain elimination. In other words, it is important to understand which, where and why people with malaria are travelling.

This study fills an important gap in knowledge and presents approaches to analysing the socio-demographic and travel profiles of malaria patients over the whole of the malaria-endemic region of southeast Bangladesh where over 90% of the cases are reported. Previous epidemiological studies on malaria in Bangladesh covered much smaller study areas in Dighinala, Khagrachhari [52], Rajasthali, Rangamati [46] and two unions (Kuhalong and Rajbila) near Bandarban town [53]. These focussed mainly on climatic, environmental and demographic factors determining malaria risk at the household level and temporal and spatial variability within subdistricts but did not explicitly measure travel.

This study provided detailed information on a large group of patients with malaria. One consistent finding across groups was that the majority of travel was local, short distance travel by predominantly men. This has been shown in other countries, e.g. Kenya, Uganda and Tanzania [37, 38].

Roughly one third of people reported travelling away from their home overnight. This group tended to travel further and for longer, and 71% reported travel to the forest at night. In addition, for the 43% that travelled away from home only during the day, most travel was for work and within the same union with only 13% to the forest, and 37% lived in the forest. Thus, much of the exposure to malaria of these daytime travellers was likely not inside the forest. In this area, as in much of Southeast Asia, many of the malaria vectors that bite during the day are known to be present outdoors near the peripheries of villages, forest fringes and fragmented forest areas [54–56]. In addition to protection of forest goers, it is thus important to also protect this group of daytime travellers from infection during their work-related travel.

Three prominent geographic patterns of travel warrant further discussion. The first was between Cox’s Bazar and Bandarban districts. This study confirmed the hypothesis that there is a travel corridor between the coastal regions and the Chittagong Hill Tracts. This has potential implications for transmission as malaria patients travel from low and moderate endemic areas such as Ramu (API = 5.67) and Lohagara (API = 0.36) subdistricts to highly endemic areas in Bandarban district. The predicted top 4 sources were all in Cox’s Bazar district. Travellers could pick up malaria in Bandarban and spread it in a receptive area such as Ramu. Predicted sinks in Bandarban included remote forest areas such as Thanchi, Ruma and Rowangchhari near the Myanmar border, and Naikhongchhari and Alikadam subdistrict bordering Cox’s Bazar. Areas with migration from areas of low transmission to high transmission and back can hinder elimination efforts. Control strategies should focus on preventative measures targeting these potential transmission routes [57, 58].

The second notable group of travel was in the north mainly from Khagrachhari to Rangamati district (16% of travel days). Dighinala in Khagrachhari was found to be a major source and sink with most travel to Sajek in Baghaichhari, with majority engaged in forest-related activities such as bamboo harvesting, and jhum cultivation again allowing for targeted focus of intervention in specific traveller groups.

The third group was from outside the study area comprising 10% of days travelled of which most travel was to Bandarban district (38%) and back (22%); a further 22% travelled to Khagrachhari district. A significant proportion of travel was from non-endemic areas to city centres such as Bandarban Sadar and Khagrachhari Sadar found to be sinks. Again, this is a similar finding to other studies, where travel is generally to neighbouring areas and major cities [37, 39]. The city centres normally have lower malaria transmission, reducing the impact of importation through these routes.

There was virtually no travel reported between the southern and northern Chittagong Hill Tracts; only 4 cases which amounted to 0.11% of travel in terms days away from residence. This is reassuring as it suggests a natural divide between two endemic regions which could be targeted separately. The separate analysis using data from this study together with parasite population genetics and cell phone records found a similar pattern of separate malaria importation routes in the north and south of Chittagong Division with high levels of genetic mixing and frequent importation into the south-west of Chittagong Division in Cox’s Bazar [44].

There was minimal foreign travel in contrast to the substantial cross-border travel between countries in the Genre Mekong Subregion (GMS) [59]. This may have been due to an under-representation of groups adjacent to the border especially in remote areas and lack of information from non-Bengali speakers in different tribal areas [60]. Also, there might be an unwillingness to report any unsanctioned travel. This has since changed in the context of recent geo-political events, and arrival of migrants to Cox’s Bazar from Myanmar [61], but this study provides a useful baseline prior to this.

Socio-demographics were a major discriminator for travel patterns in terms of extent of travel and where people travelled to within Chittagong Division. Travellers with high potential to spread malaria were military, government personnel and border police visiting malaria-endemic areas. Military personnel visiting form outside Chittagong Division and border police residing in Bandarban and Cox’s Bazar travelled the furthest. Similarly, business professionals travelled long distances especially from outside the study areas to Bandarban, and from Chittagong to Bandarban. A previous study in Tripura, India, identified military personnel along the India-Bangladesh border to be at higher risk of contracting malaria due to lower immunity [20]. These groups should be targeted to mitigate transmission of malaria through travel.

Another key group potentially increasing transmission through travel were individuals engaging in forest activities who tended to travel further, including travelling to deep forest areas, along with longer overnight stays. The top four destination unions for forest travellers were Alikadam, Lama, Sarai and Rupshipara, all situated in Bandarban district. Males of working age (25–49 years) comprised the largest group of forest visitors, and reasons for travelling to the forest included cutting wood, jhum cultivation, plantation, bamboo collection and miscellaneous daily labourer activities. Jhum workers can be found in both the northern and southern part of Chittagong Hill Tracts, and are a particularly important group to target, as they have been found to be asymptomatic carriers of malaria, with the risk of contracting malaria increasing in other household members who do not engage in jhum cultivation [25, 33]. Similar findings in the GMS implicate men of working age to be at higher risk of contracting malaria through occupational activities such as working in the forest. Somboon [62] noted in an observational study at the border of Thailand and Myanmar that increased forest activity in the dry season and also staying overnight in the rainy season in the paddy fields resulted in increased risk of contracting malaria. Similarly, Jarai youth near the Cambodia-Vietnam border residing overnight in forest for work were identified as a risk group due to low uptake of preventative measures such as bed nets [63]. Interestingly, in Bangladesh, no association was found with use of bed nets and reduction in risk of forest malaria in jhum workers and was attributed to the presence of exophilic mosquitoes [60].

Housewives and children aged less than 15 years were less likely to travel (*p* < 0.001). Students and forest dwellers and people who did not visit the forest travelled mainly within the same union. It can be inferred from this that children, housewives and forest dwellers with malaria are more likely to have contracted malaria locally, and thus local measures should be the focus of malaria control for these demographic groups. Previous studies in the Chittagong Hill Tracts investigating local population at risk of malaria in rural villages have identified children under 10 years, forest dwellers, and also inhabitants living in fragmented forest, village peripheries or near water bodies [64, 65], as specific risk groups. The finding that women and children do not travel much in Bangladesh is in direct contrast to another large travel survey conducted across Mali, Burkina Faso, Zambia and Tanzania where women travelling with children were identified as a consistent group across all four countries [66]. However, the malaria status of participants in this African study was unknown and the geographical context is very different from Bangladesh with a much larger study area with more long-distance travel. A recent study conducted in Swaziland in an area of low malaria transmission looked at travellers stratified by their malaria status and found a gender bias, with males of working aged 25–44 years to be at higher risk of contracting malaria when crossing international borders [67].

When engaging in regular non-work travel, the majority of the study participants cited market and social reasons such as visiting a relative. Although 93% of this subgroup reported travel outside their residential union, the median distance of travel was only 17 km, as travel tended to be in neighbouring unions and subdistricts. Similar findings were noted in travel surveys conducted in rural areas around Lake Victoria, Kenya [37], where if individuals travel they went to neighbouring districts or those including a major city with the primary motivation of visiting family or friends.

Limitations were noted in the study. For example, women are less likely to have means to travel to access health care or will seek care from a local healer before coming to a diagnostic facility [68, 69], which could potentially result in lower recruitment of women. There was also an under-recruitment in remote forested areas such Rowangchhari, Ruma and Thanchi despite them having high API due to remoteness. In the CHT, people seek healthcare from either health facilities or, particularly in more remote areas, through home visits by BRAC (a local NGO consortium). As recruitment for this study was at health facilities, patients treated only at home were not included in the study. Despite anecdotal information from the NMEP that there is frequent cross-border migration, the travel survey did not reflect this and could be an underestimate due to cross-border migration occurring in remote areas and patients perhaps not wanting to report such activities. Information on overnight travel was only conducted in a separate section asking about travel to the forest, long-distance trips, but not in regard to regular daily work as it was assumed from the pilots that very little of this would be overnight. A compromise had to be struck between the level of detail of the questionnaire and its length, to enable health workers to administer this questionnaire with minimal training. Another limitation of the study is the lack of a control group for the travel analysis. Demographics were compared with the census as a surrogate for other data. Travel patterns of healthy non-malaria infected patients, or patients presenting with a fever but testing negative, are not known. Inferences about likely locations of malaria transmission, importation vs local infection, and therefore sources and sinks of malaria are necessarily estimates. This may be improved by further development of methods and modelling together with incorporation of additional epidemiological and genetic data [44]; however, the methods in this paper used data which are relatively easy for an NMCP to collect. The survey tool captured information about numbers of nights travelled separately to the number of days. In this region, some of the local malaria vectors bite during the day whereas others bite more in the evening or overnight. With more detailed information about the contribution of different vectors to transmission and their behaviour in Bangladesh, it might be possible to make inferences about the relative importance of daytime versus nighttime travel for transmission. However, such information was not available. The smallest unit of analysis was the union, and the subdistrict level when comparing results to national incidence data, as more detailed maps and village-level surveillance data were not available. Most of the travel was found to be within the same union. Work is currently underway to collect village-level location information to further improve the resolution of the results.

## Conclusion

The epidemiology of travel patterns of malaria patients in southeast Bangladesh is complex with widespread temporal and spatial heterogeneity, presenting unique challenges for malaria control and needing targeted spatial interventions. Travel contributes to this spatial heterogeneity and complexity. Through the use of a simple travel questionnaire administered by healthcare staff at a wide range of facilities, this study identifies key routes of travel for malaria patients, and differences in connectivity for different traveller groups. The approach demonstrated here provides a framework for identifying key travellers’ groups and their origins and destinations of travel in combination with knowledge of local epidemiology to inform malaria control and elimination efforts.

## Supplementary information

Additional file 1: Supplementary information and analysis.

## Abbreviations

ACT: Artemisinin combination therapy
API: Annual Parasite Incidence
BMG: Bangladesh Mobility Genotype
CHT: Chittagong Hill Tracts
GMS: Genre Mekong Subregion
NEMP: National Malaria Elimination Programme
*P. falciparum*: *Plasmodium falciparum*
*P. vivax*: *Plasmodium vivax*
