# Supplementary material for: Mapping the travel patterns of people with malaria in Bangladesh
Source: BMC Med. 2020 Mar 4;18:45. doi: 10.1186/s12916-020-1512-5 (PMC7055101; doi:10.1186/s12916-020-1512-5)
Supplement: Supplementary file 1 — Supplementary information and analysis. [file 12916_2020_1512_MOESM1_ESM.pdf]

Mapping the travel patterns of people with malaria in Bangladesh

SUPPLEMENTARY INFORMATION

Diagnostics

Table S1: RDT brands used for diagnosis by the NMEP

Year	RDT Brand	Infections diagnosed
2013-2014	First Response Malaria pLDH/HRP Combo Card Test	*P.falciparum*, non-*P. falciparum* and mixed
Mid 2015 till August 2016	One Step Malaria HRP2 (*P. falciparum*)/pLDH (Pan) Antigen RDT	*P.falciparum*, non-*P. falciparum* and mixed
August 2016 onwards	Care Start P. falciparum/P. vivax (*Implemented as RDT of choice but information on how quickly roll-out occurred was not available)	*P.falciparum*, *P. vivax* and mixed

Temporal pattern of enrolment

Forty-five percent of cases were recruited during the monsoon in 2016, from May up to the end of the study. There was a smaller peak during the monsoon from August till October 2015. This broadly followed the national seasonal peaks in incidence reported to the NMEP, with a slower uptake at the beginning of the study period as expected.

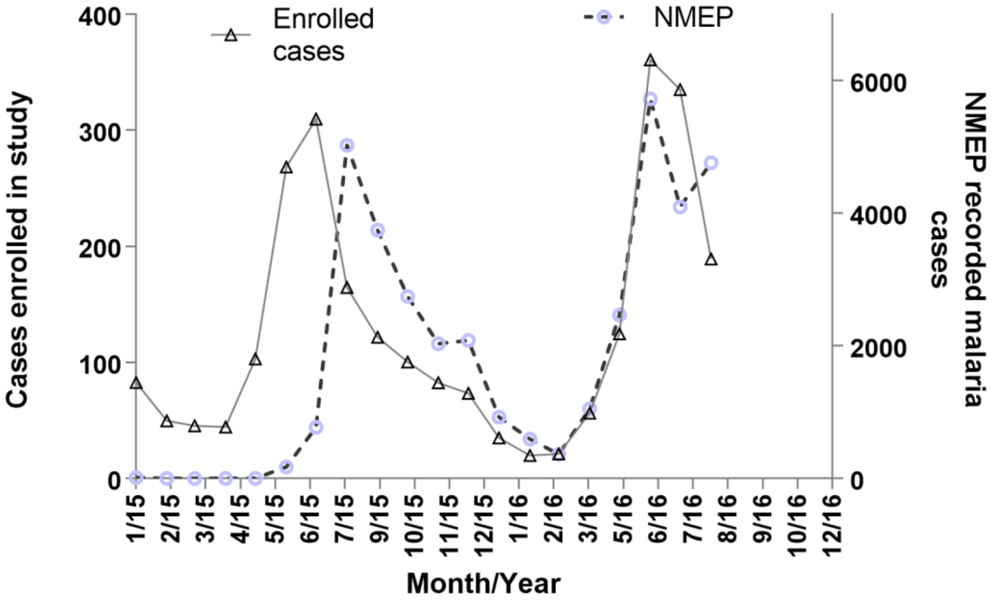

Figure S1: Monthly case recruitment during the study period.

Completeness of data

A hundred percent of the cases had a recorded residential address, gender and age. Eight cases had missing occupation information.

Recruitment vs incidence

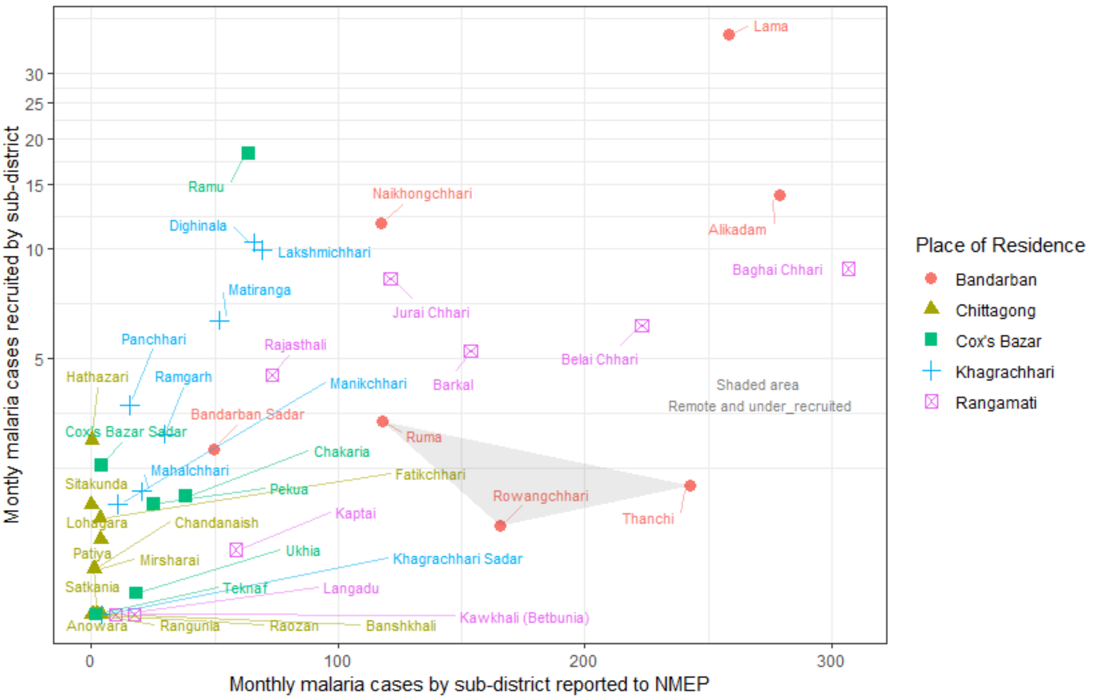

Figure S2: Monthly recruitment to study vs NMEP monthly incidence by sub-district Ruma,

Rowangchhari, Thanchi subdistricts (upazilas) (coloured in grey) in Bandarban district (red) had lower levels of recruitment for the study compared to surveillance data. Ramu, the site of a concurrent malaria research study, had a higher proportion of study recruitment than the proportion of incidence reported to the NMEP.

Demographics: Age population pyramid: Study compared to census

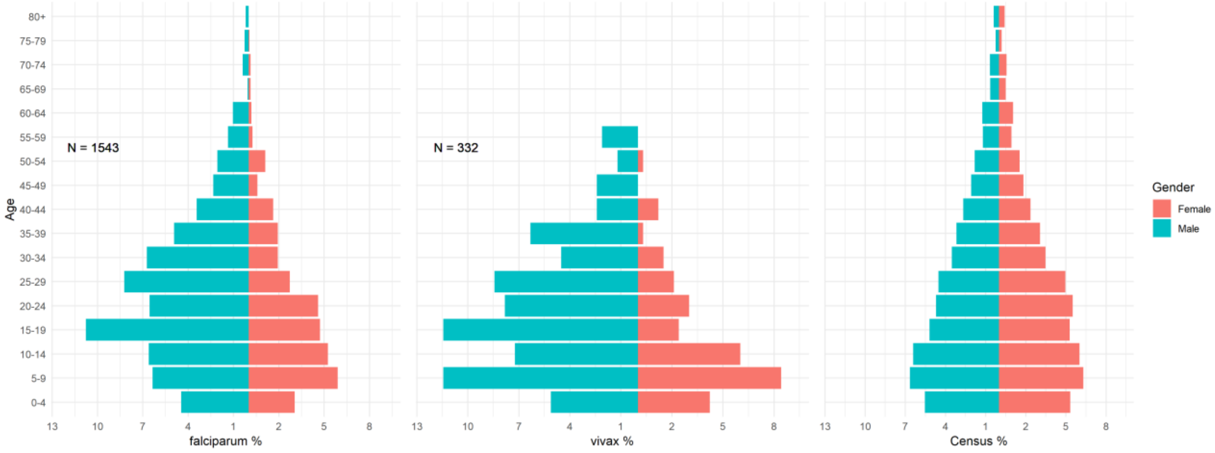

Figure S3: Age and gender population pyramid of enrolled patients by species and census study area in Chittagong Division.

Plasmodium species distribution

Species distribution by district

Table S2: Monthly cases recruited during the study period by district of residence

District	Pf (n)	%	Pv (n)	%	Mixed PfPv (n)	%
Bandarban	698	45.3	213	64.2	122	56.7
Khagrachhari	359	23.3	17	5.1	40	18.6
Cox's Bazar	218	14.1	80	24.4	24	11.2
Rangamati	196	12.7	14	4.2	23	10.7
Chittagong	62	4.0	2	0.6	4	1.9
Other	9	0.6	5	1.5	2	0.9

The northern districts of Khagrachhari and Rangamati had proportionally less vivax compared to the southern districts This was different in the NMEP surveillance data where Bandarban and Rangamati comprised the highest proportion of vivax cases at 52 and 30% (southern and northern districts), followed by Cox Bazar (8%), Khagrachhari (7%) and Chittagong (1%). As discussed earlier, Rangamati only contributed cases in the second year of the study.

Species distribution by age and gender

Table S3: Age and gender distribution of enrolled patients by Plasmodium species

	Pf		Pv		Mixed PfPv		P Value
	n	%	n	%	n	%	
Sex							0.118
Female	528	34.2	98	29.5	73	34.0	
Male	1015	65.8	234	70.5	142	66.0	
Age group (years)							< 0.001
Age < 1 year	22	1.4	4	1.2	3	1.4	
Age 1 – 4	136	8.8	42	12.7	27	12.6	
Age 5-14	368	23.8	103	31.0	56	26.0	
Age 15+	1017	65.9	183	55.1	129	60.0	
Age (median, Quantile)	20	(12-32)	18	(8-28)	20	(9.5-30)	

The proportion of children under 15 was higher for P. vivax compared to P. falciparum (P < 0.001).

Species distribution by occupation and travel to forest

There were no significant differences between Plasmodium species for occupation or reported travel to the forest (Table S4 and Table S5). The top four occupations were predominant in the hill-tract districts, whereas in Chittagong there was a wider diversity in occupations, including a higher proportion of military personnel. Only 16 cases were reported as resident outside the study catchment area, 14 of these were in the military. For women, 30% were housewives, followed by students (22%) and children (24%). Out of the group who gave working in the forest as an occupation, 12% did not visit the forest. These included cases working in jhum cultivation (slash and burn farming, usually in the dense forest).

Table S4: Occupation by malaria species and gender of enrolled patients

Occupation	Pf		Pv		Mixed PfPv		P Value	Male		Female		P Value
	n	%	n	%	n	%		n	%	n	%	
Farming	352	22.9	48	14.5	61	28.3	0.23	389	28.0	72	10.3	0.25
Student	272	17.6	79	23.8	39	18.1		225	16.2	165	23.6	
Forestry	269	17.4	52	15.7	22	10.2		286	20.6	57	8.2	
Child	233	15.1	62	18.7	46	21.4		186	13.4	155	22.2	
Housewife	181	11.7	22	6.6	19	8.8		0	0	222	31.7	
Labourer	59	3.8	11	3.3	8	3.7		74	5.3	4	0.6	
Business	39	2.5	13	3.9	7	3.3		56	4.0	3	0.4	
Military	43	2.8	9	2.7	7	3.3		59	4.2	0	0	
Jobless	21	1.4	12	3.6	1	0.5		26	1.9	8	1.1	
Teacher	20	1.3	8	2.4	1	0.5		25	1.8	4	0.6	
Carpenter	18	1.2	4	1.2	1	0.5		23	1.6	0	0.0	
Other	36	2.3	12	3.6	3	1.4		42	3.0	9	1.3	

Table S5: Species distribution by travel to forest or whether patients lived in the forest

	Pf		Pv		Mixed PfPv		P Value
Forest Status	n	%	N	%	n	%	
							0.6
Dweller	484	31.4	99	29.8	74	34.4	
Visitor	498	32.3	117	35.3	51	23.7	
Never Visited	561	36.4	116	34.9	90	41.9	

Travel

Travel Distribution – from residence to destination

Table S6: Distribution of days travelled by purpose of reported travel and administrative division

**Travel purpose**	**Total days travelled over 2 months** (absolute numbers)	**% of travel days by administrative level**				
		Within Union	Inter union within same subdistrict	Inter subdistrict within same district	Inter district within same division	Outside Chittagong Hill Tracts
**Work**	71,325	87	5	2	5	1
**To forest**	12,859	47	10	8	34	1
**Frequent non work travel**	2,633	57	16	12	11	4
**Infrequent non work travel**	866	37	14	8	28	13
**All Travel within Bangladesh**	87,683	80	6	3	10	1

Table S7: Distribution of nights travelled by purpose of reported travel and administrative divisions

**Travel purpose**	**Total nights travelled over 2 months** (absolute numbers)	**% of travel nights by administrative level (not including work)**				
		Within Union	Inter union within same subdistrict	Inter subdistrict within same district	Inter district within same division	Outside Chittagong Hill Tracts
**To forest**	8,772	32	11	10	45	2
**Frequent non work travel**	1,384	48	12	18	14	8
**Infrequent non work travel**	661	35	13	7	29	16
**All Travel within Bangladesh**	**10,817**	34	11	11	41	3

Table S8 and Table S9 highlight the top 12 travel origin-destination combinations cited by days and nights respectively. The spatial resolution is at the union level. Figure S4 shows the geographic distribution of nights spent away from the place of residence, discussed in more detail in the main paper. Figure S5 and S6 summarizes inter-district travel, again discussed in the main paper.

Table S8: Top 12 origin-destination combinations by days travelled in last two months

Residence			Destination			Travel	
District	Subdistrict	Union	District	Subdistrict	Union	Days	%
Bandarban	Lama	Lama	Bandarban	Lama	Lama	6,213	7
Bandarban	Alikadam	Alikadam	Bandarban	Alikadam	Alikadam	5,319	6
Bandarban	Lama	Sarai	Bandarban	Lama	Sarai	4,416	5
Bandarban	Lama	Rupshipara	Bandarban	Lama	Rupshipara	4,064	5
Cox's Bazar	Ramu	Garjania	Cox's Bazar	Ramu	Garjania	3,018	3
Bandarban	Lama	Gajalia	Bandarban	Lama	Gajalia	2,854	3
Khagrachhari	Dighinala	Babuchhara	Khagrachhari	Dighinala	Babuchhara	2,706	3
Rangamati	Baghaichhari	Sajek	Rangamati	Baghaichhari	Sajek	2,588	3
Bandarban	Naikhongchhari	Naikhongchhari	Bandarban	Naikhongchhari	Naikhongchhari	2,304	3
Bandarban	Alikadam	Chokhyong	Bandarban	Alikadam	Chokhyong	2,297	3
Cox's Bazar	Ramu	Kachhapia	Cox's Bazar	Ramu	Kachhapia	2,006	2
Khagrachhari	Lakshmichhari	Lakshmichhari	Khagrachhari	Lakshmichhari	Lakshmichhari	1,775	2
Others						48,123	55
Total days travelled						87,683	100

Table S9: Top 12 origin-destination combinations by nights travelled in last two months

Residence			Destination			Travel	
District	Subdistrict	Union	District	Subdistrict	Union	Nights	%
Bandarban	Alikadam	Alikadam	Bandarban	Alikadam	Alikadam	593	5
Cox's Bazar	Ramu	Kachhapia	Bandarban	Naikhongchhari	Dochhari	544	5
Cox's Bazar	Ramu	Khuarkhop	Bandarban	Alikadam	Alikadam	540	5
Bandarban	Lama	Sarai	Bandarban	Lama	Sarai	397	4
Khagrachhari	Dighinala	Babuchhara	Rangamati	Baghaichhari	Sajek	390	4
Bandarban	Lama	Lama	Bandarban	Lama	Lama	376	3
Rangamati	Baghaichhari	Sajek	Rangamati	Baghaichhari	Sajek	330	3
Cox's Bazar	Ramu	Garjania	Bandarban	Naikhongchhari	Dochhari	311	3
Cox's Bazar	Ramu	Garjania	Bandarban	Alikadam	Alikadam	301	3
Khagrachhari	Dighinala	Babuchhara	Khagrachhari	Dighinala	Babuchhara	277	3
Cox's Bazar	Ramu	Garjania	Bandarban	Naikhongchhari	Baishari	214	2
Bandarban	Alikadam	Chokhyong	Bandarban	Alikadam	Alikadam	189	2
Others						6,355	59
Total nights travelled						10,817	100

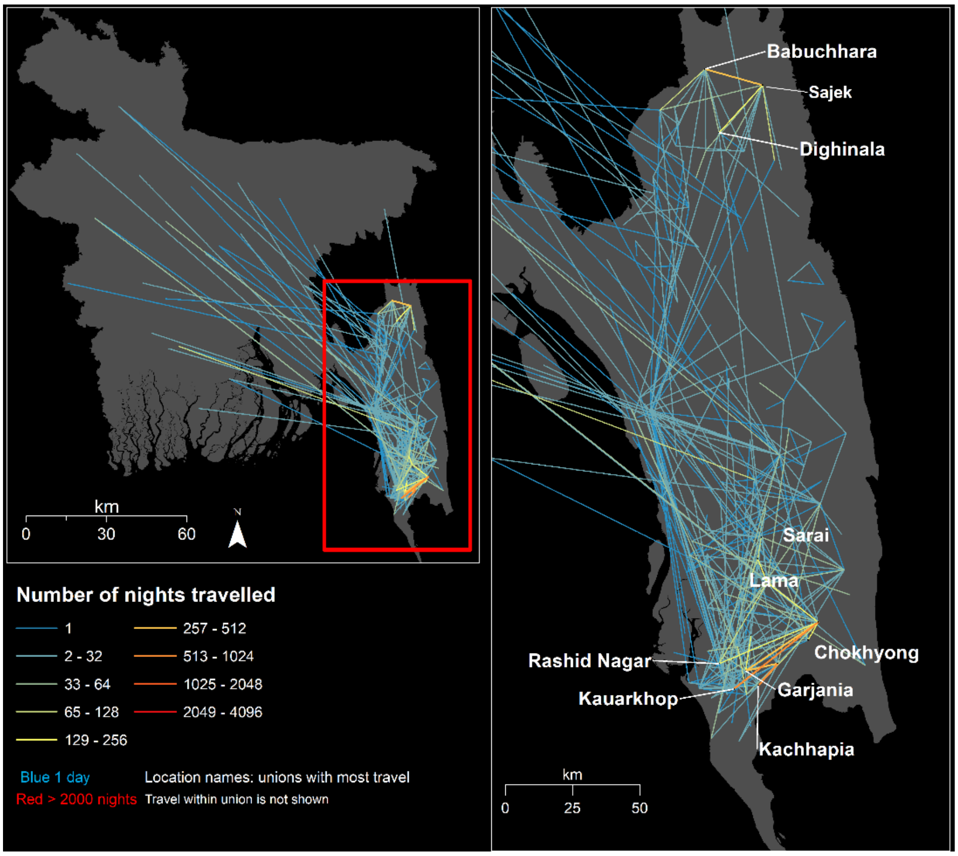

Figure S4: Nights away from residence and destination over the past two months

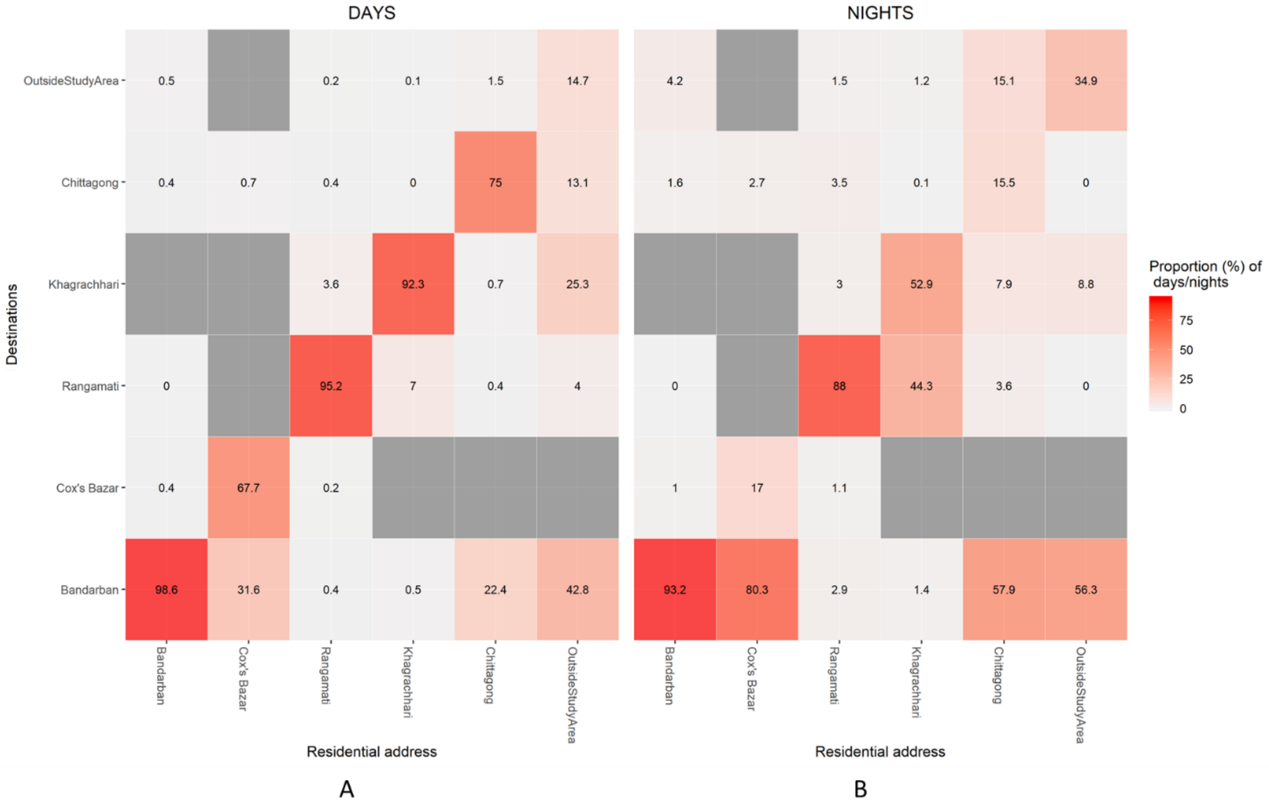

Figure S5 (a) Proportion of travel days and (b) nights from residence to different travel destinations at district level. Columns sum to 100%.

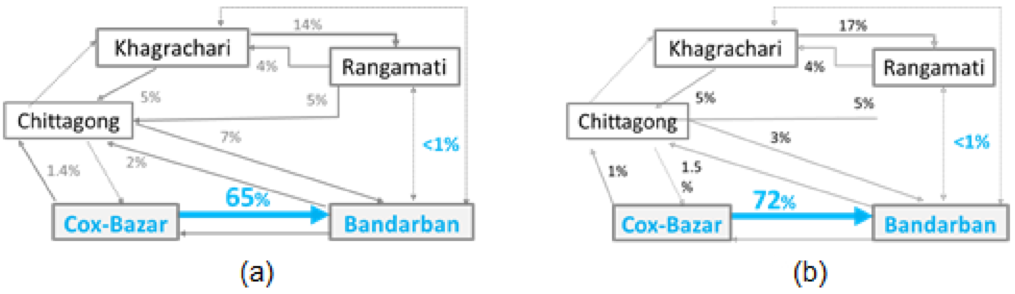

Figure S6: Inter-district travel by (a) days (b) nights

Travel distance

The distances travelled from place of residence to the study site (place of diagnosis) were exponentially distributed (Figure S7). The overall geographic pattern of travel for nights away from home was similar to that for days of travel (Figure S8). Proportionally, people travelled further and longer if spending nights away as compared to days. There was a J-shaped distribution with most travel within 100 km of place of residence (note y axis is log scale). The within-union distance was estimated to be the 95% percentile of nearest neighbour distance at just under 5km.

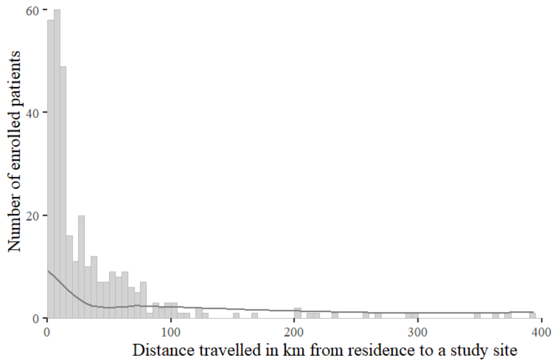

Figure S7: Histogram of cases travelling from residence to study-site by distance in km

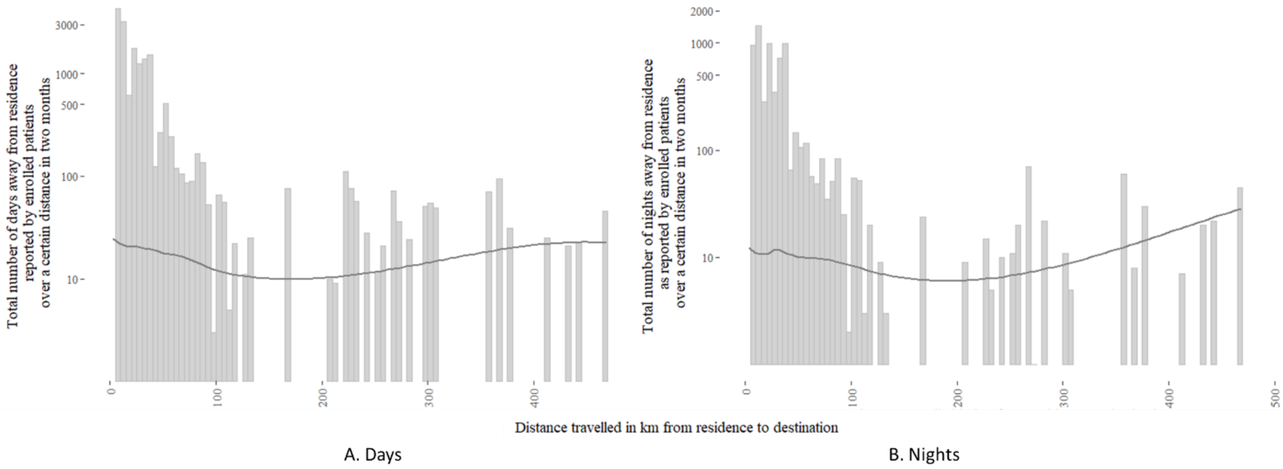

Figure S8: Distance travelled from origin to destination by a) number of days and b) number of nights

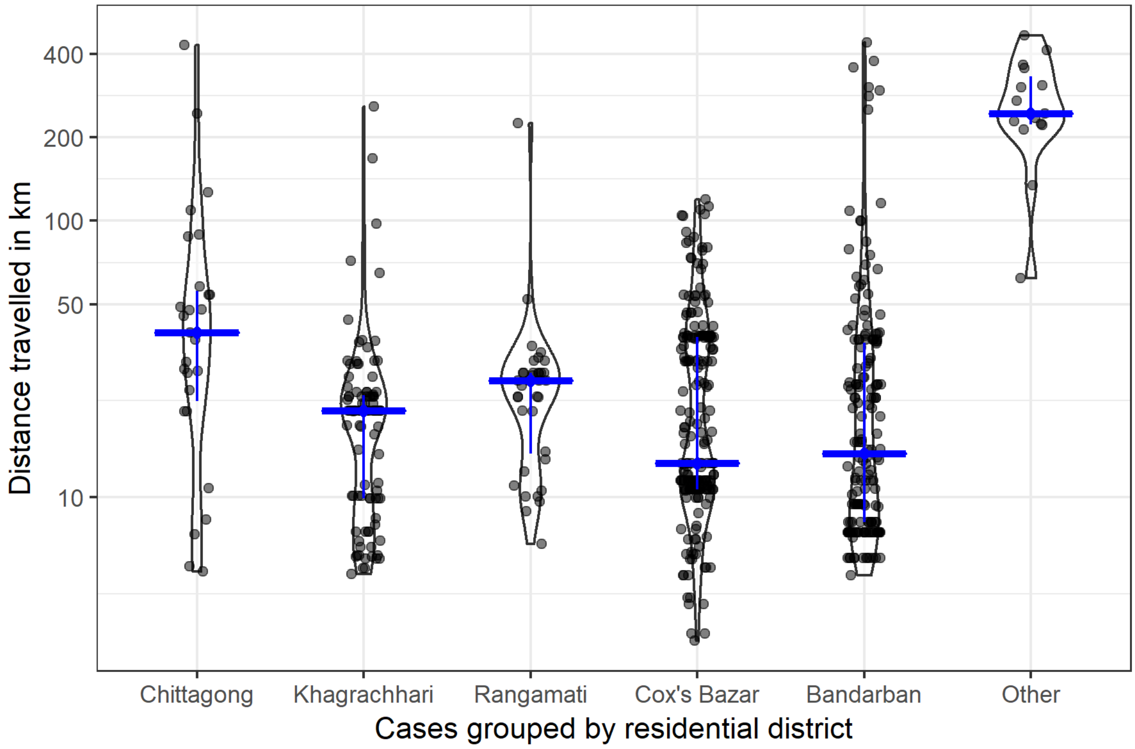

Figure S9: Distance travelled in km from residential union to destination union grouped by district of residence. Median distance shown in horizontal cross bars and interquartile range as vertical blue lines. ‘Other’ is resident in any other district outside the study area.

Malaria sources and sinks

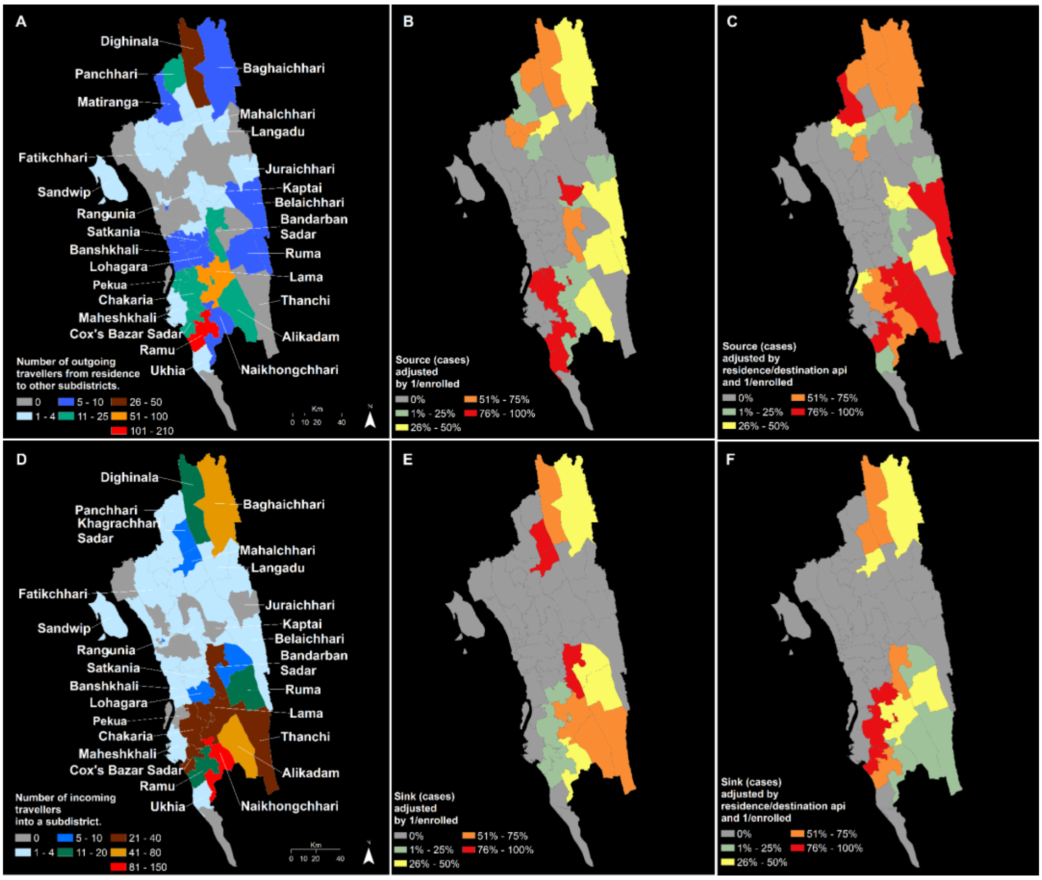

Figure S10: Sources and sinks (not accounting for duration): From left to right: (a) outgoing traveller numbers, (b) quartiles of ranked sources method 1, (c) quartiles of ranked sources method 2, (d) incoming traveller numbers, (e) quartiles of ranked sinks method 1, (f) quartiles of ranked sinks method 2.

Table S10: Top predicted source subdistricts.

**Predicted sources (by cases) adjusted for enrolment (Method 1)**					
District	Subdistrict	Outgoing	Enrolled	Origin API	Quartile
Cox's Bazar	Pekua	13	12	5	Top 25%
Cox's Bazar	Chakaria	20	21	2	Top 25%
Cox's Bazar	Ramu	206	254	6	Top 25%
Cox's Bazar	Ukhia	4	8	2	Top 25%
Rangamati	Kaptai	3	6	20	Top 25%
Khagrachhari	Dighinala	46	135	13	50-75%
Khagrachhari	Panchhari	14	41	5	50-75%
Bandarban	Bandarban Sadar	12	31	13	50-75%
Khagrachhari	Ramgarh	4	28	9	50-75%
Khagrachhari	Manikchhari	1	8	4	50-75%
Bandarban	Alikadam	25	195	133	25-50%
**Predicted sources adjusted for enrolment and API (Method 2)**					
District	Subdistrict	Outgoing	Enrolled	Origin API	Quartile
Rangamati	Belaichhari	5	43	169	Top 25%
Bandarban	Alikadam	25	195	133	Top 25%

Bandarban	Lama	53	574	57	Top 25%
Cox's Bazar	Ramu	206	254	6	Top 25%
Khagrachhari	Matiranga	7	89	9	Top 25%
Cox's Bazar	Chakaria	20	21	2	Top 25%
Khagrachhari	Panchhari	14	41	5	50-75%
Rangamati	Baghaichhari	7	97	67	50-75%
Khagrachhari	Lakshmichhari	3	99	55	50-75%
Khagrachhari	Dighinala	46	135	13	50-75%
Bandarban	Naikhongchhari	10	164	43	50-75%

Table S11: Top predicted sink subdistricts

**Predicted sinks (by cases) adjusted for enrolment (Method 1)**					
District	Subdistrict	Incoming	Enrolled	API	Quartile
Bandarban	Bandarban Sadar	36	31	13	Top 25%
Khagrachhari	Khagrachhari Sadar	9	3	1	Top 25%
Khagrachhari	Mahalchhari	5	13	9	Top 25%
Bandarban	Alikadam	73	195	133	50-75%
Bandarban	Thanchi	25	18	241	50-75%
Khagrachhari	Dighinala	18	135	13	50-75%
Bandarban	Lama	33	574	57	50-75%
Bandarban	Rowangchhari	7	14	135	25-50%
Bandarban	Ruma	17	37	89	25-50%
Rangamati	Baghaichhari	44	97	67	25-50%
Bandarban	Naikhongchhari	109	164	43	25-50%
**Predicted sinks by cases adjusted for enrolment and API (Method 2)**					
Cox's Bazar	Cox's Bazar Sadar	29	23	0	Top 25%
Cox's Bazar	Chakaria	22	21	2	Top 25%
Chittagong	Lohagara	7	8	0	Top 25%
Khagrachhari	Khagrachhari Sadar	9	3	1	50-75%
Bandarban	Bandarban Sadar	36	31	13	50-75%
Khagrachhari	Dighinala	18	135	13	50-75%
Cox's Bazar	Ramu	13	254	6	50-75%
Bandarban	Ruma	17	37	89	25-50%
Bandarban	Lama	33	574	57	25-50%
Khagrachhari	Mahalchhari	5	13	9	25-50%
Rangamati	Baghaichhari	44	97	67	25-50%

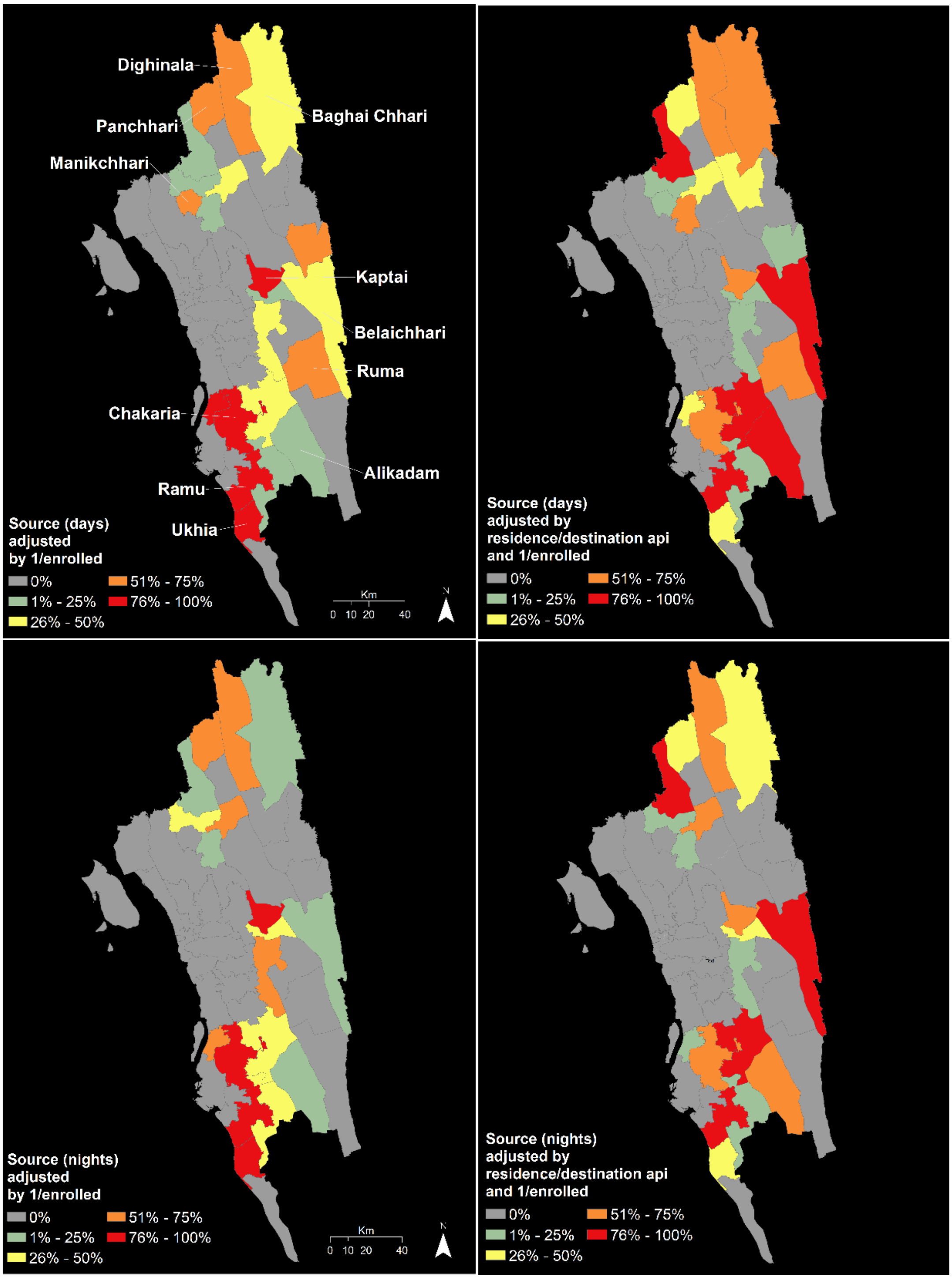

Figure S11: Predicted sources: top panel – travel days (a) Method 1 and (b) Method 2, bottom panel – travel nights (c) Method 1 and (d) Method 2.

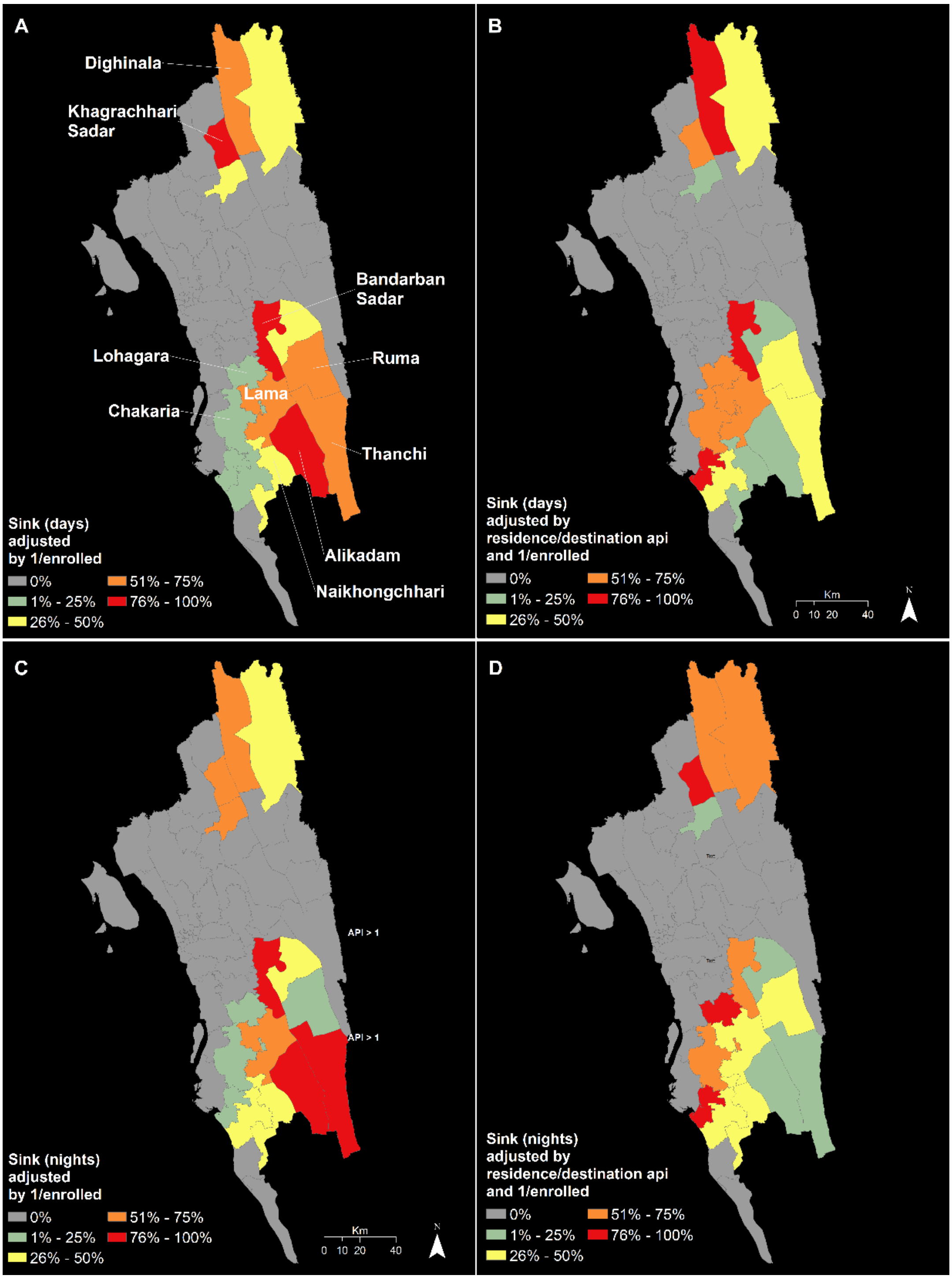

Figure S12: Predicted sinks: top panel – travel days (a) Method 1, and (b) Method 2, and bottom panel – travel nights (c) Method 1 and (d) Method 2.

Ramu and neighbouring subdistricts in Cox's bazar district were predicted to be sources. Bandarban Sadar, Alikadam, and Thanchi, remote forested areas, were sinks. Dighinala was both a source and sink in the northern Chittagong Hill Tracts.

Travel and demographics

Travel, age and gender

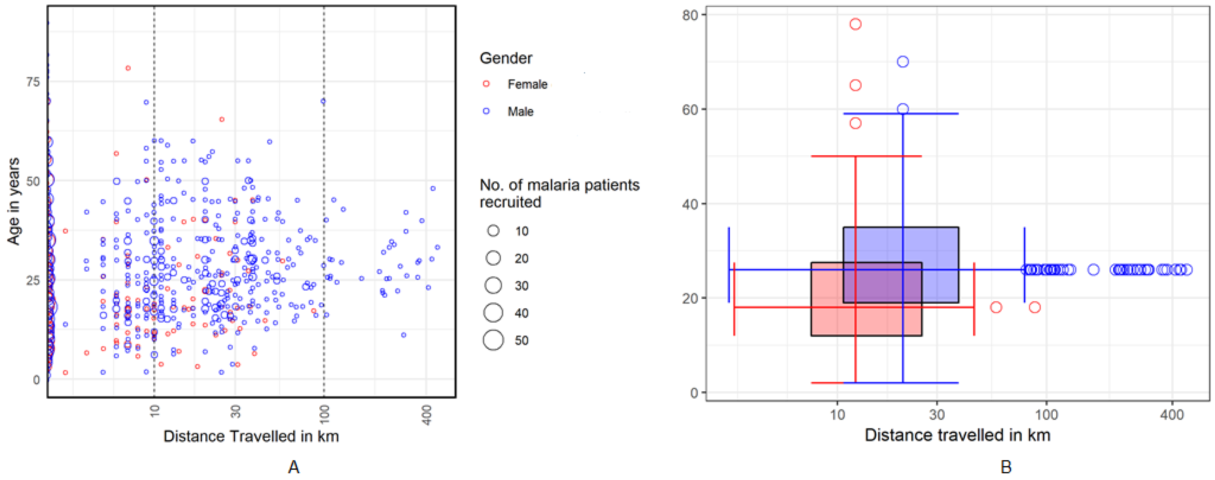

Figure S13: (a) Distance (km) travelled by enrolled cases by age and gender (b) summarised as median age and distance along with their interquartile range (note within-union travel excluded).

Table S12: Number of malaria cases that travelled by gender and distance.

	Enrolled cases	Travelled (% of enrolled)	25th	50th (median)	75th	95th
			Percentile of distance travelled in km			
Male	1391	1225 (89%)	11	21	38	184
Female	699	406 (81%)	7	12	25	38

Travel and occupation

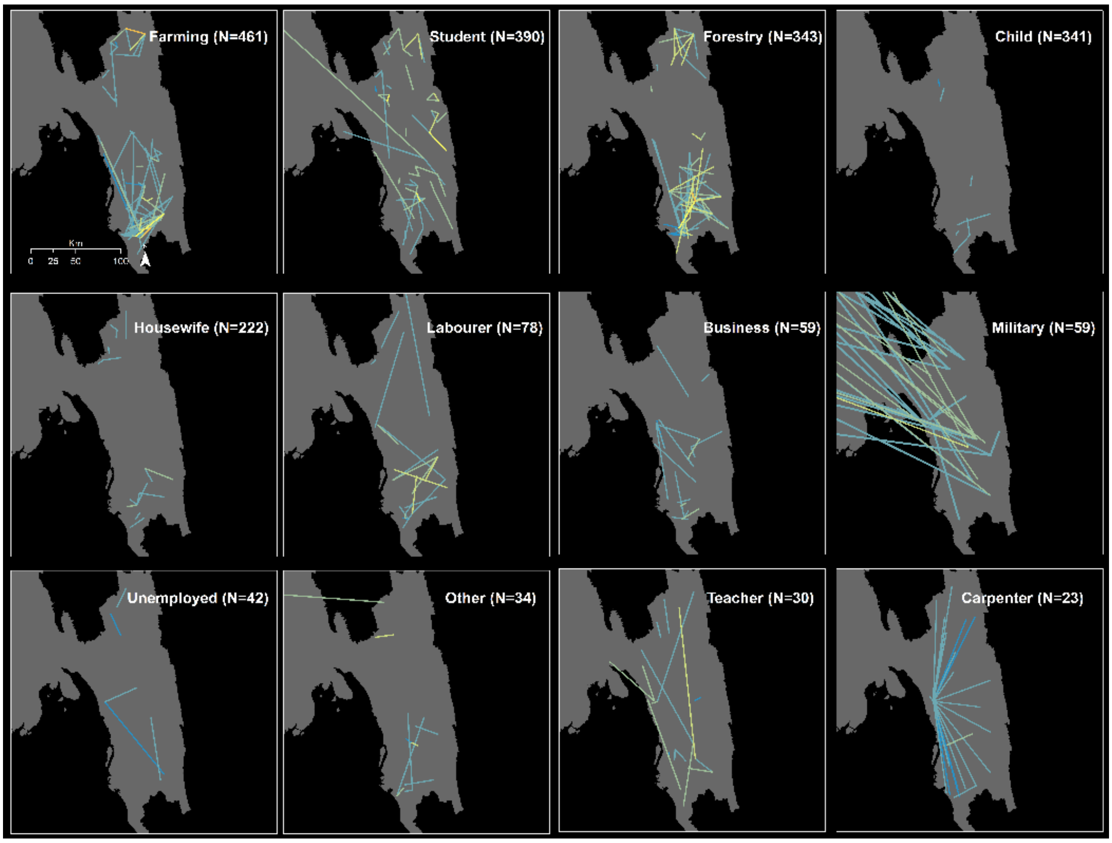

Figure S14(a): Number of days travelled between residence and destination over two months by occupation. Note the panels are ordered in ascending order of number of cases enrolled by occupation.

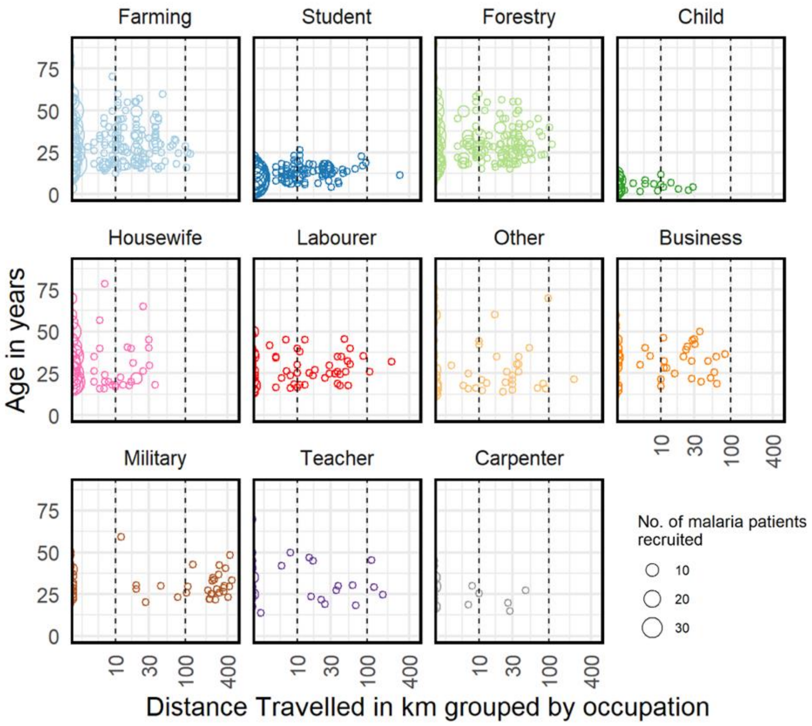

Figure S14 (b): Distance (km) travelled by enrolled cases by occupation and age

Associations between travel and demographics

An exploratory multiple correspondence analysis (MCA) analysis was performed on age, gender, occupation and forest status to identify factors related to travel (Figure S5). There was a clustering of factors by the groups "travel" versus "do not travel". Children under 4 years of age and housewives travelled very little even within the same union. Of note, child was reported as an occupation with the median age of 8 years if they went elsewhere for work or school. The median age for a child who did not go to school was 5 years. Students has a median age of 11 years and a maximum age of 26 years and were mutually exclusive from children in the reported occupation.

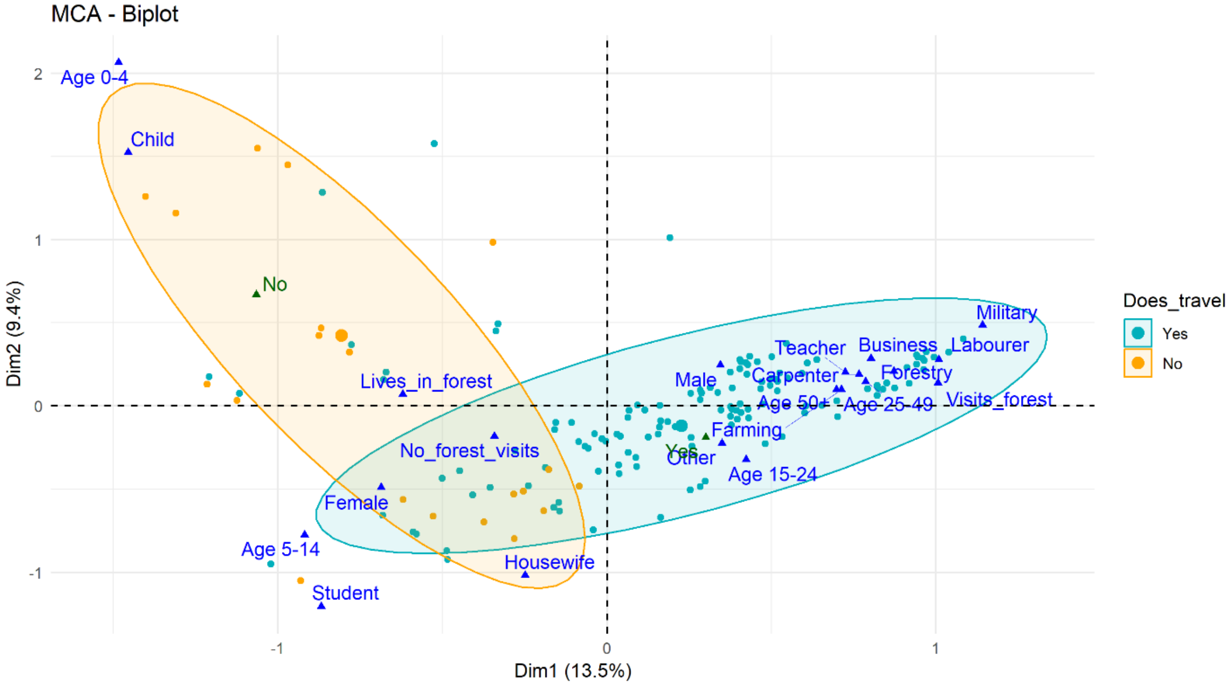

Figure S15(a): Travel in relation to demographics (age, gender, occupation and forest travel status)

This analysis was then modified to group travel by extent of travel i.e. travel outside a union, a subdistrict or a district. (Figure S15b). Again, children under 4 years of age were not part of any clusters as they travelled very little. There was overlap between the three groups with certain groups restricted to travel within smaller administrative divisions. For example, women, children age 5-14 years and students travelled within a subdistrict but not further. Both groups who did not visit the forest and people who lived in the forest did not travel beyond their own residential district. All other demographic groups travelled outside their district with forest visitors, military and business occupation most associated with travel beyond district.

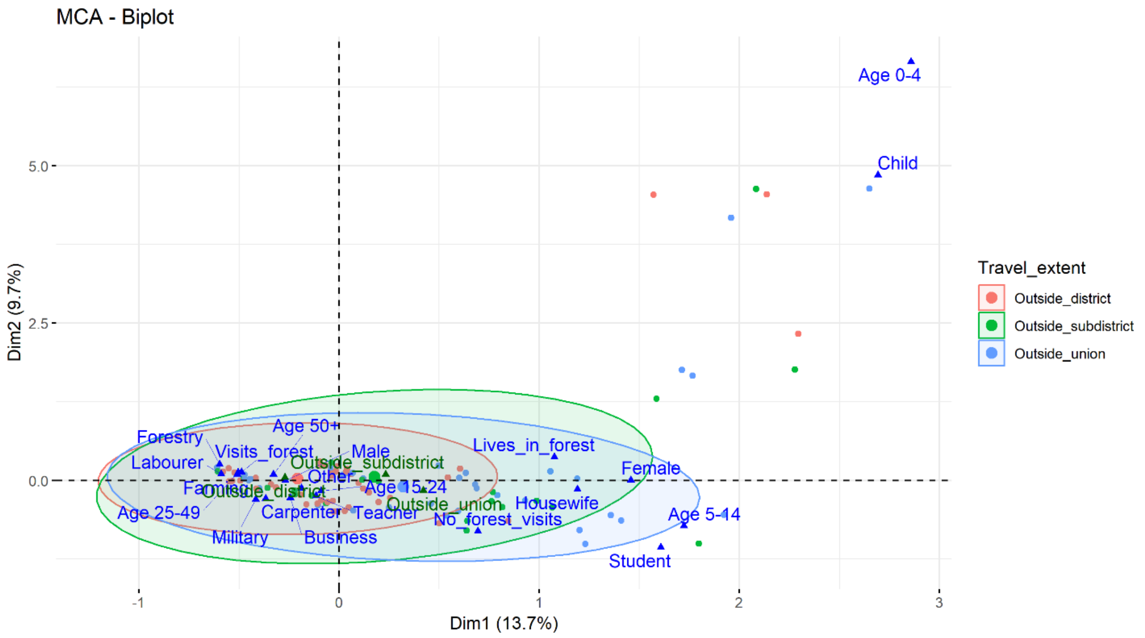

Figure S15 (b): MCA analysis to group factors in relation to extent of travel: travel outside a union, subdistrict or district.

A univariate analysis was done by whether a group travels or not, and the extent of their travel, i.e. outside the union, subdistrict or district, for the following demographic factors – age, occupation, travel to forest and gender. The women, housewives, children aged under 5 years, people who live in the forest and who did not visit the forest were less likely to travel. The pattern is similar for travel outside the union, sub-district and district with an additional group – children aged 5-14 years - significant when considering travel beyond the union and subdistrict.

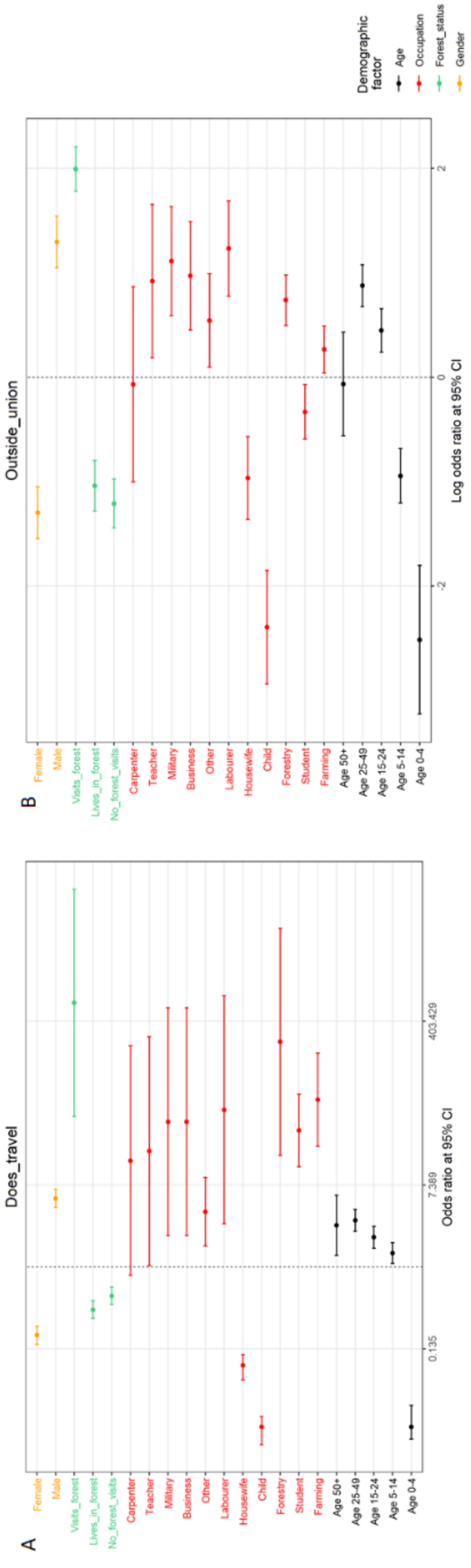

Figure S16: Univariate log odds in relation to whether a group (a) reported travel or not, (b) outside union, (c) outside subdistrict, and (d) outside district

Table S13: Odds ratios of demographic factors in relation to travel from top quartile of source areas (Method 1) from multivariate analysis.

	**Outside Upazila**			**Outside District**		
Predictors	Odds Ratios	CI	p	Odds Ratios	CI	p
Age 0-4	0.43	0.09 – 1.97	0.275	0.30	0.05 – 1.91	0.202
Age 5-14	1.03	0.30 – 3.53	0.965	1.20	0.33 – 4.32	0.780
Age 15-24	2.16	0.81 – 5.80	0.125	1.92	0.70 – 5.25	0.204
Age 25-49	2.70	1.02 – 7.14	**0.045**	2.64	0.98 – 7.10	0.054
Male	3.84	1.86 – 7.92	**<0.001**	4.54	2.00 – 10.32	**<0.001**
Forest Dweller	0.49	0.28 – 0.88	**0.017**	0.36	0.19 – 0.66	**0.001**
Student	0.77	0.25 – 2.39	0.654	0.67	0.19 – 2.40	0.540
Farming	1.31	0.56 – 3.08	0.530	1.44	0.57 – 3.63	0.435
Forestry	2.79	1.16 – 6.72	**0.022**	3.05	1.19 – 7.82	**0.020**
Labourer	2.90	0.91 – 9.27	0.073	3.52	1.07 – 11.60	**0.038**
Other	5.18	1.40 – 19.26	**0.014**	4.56	1.26 – 16.49	**0.021**
Business	7.06	0.73 – 68.52	0.092	4.71	0.72 – 30.78	0.106
Military	0.27	0.02 – 2.97	0.284	0.38	0.03 – 4.28	0.430
Observations	471			471		

Proportion of travel from residential district to other districts

The proportion of travel by different metrics is represented by heatmaps assessing the proportion of travel from residential district to destination for different demographic groups. The maps for travel by day for key groups are shown below.

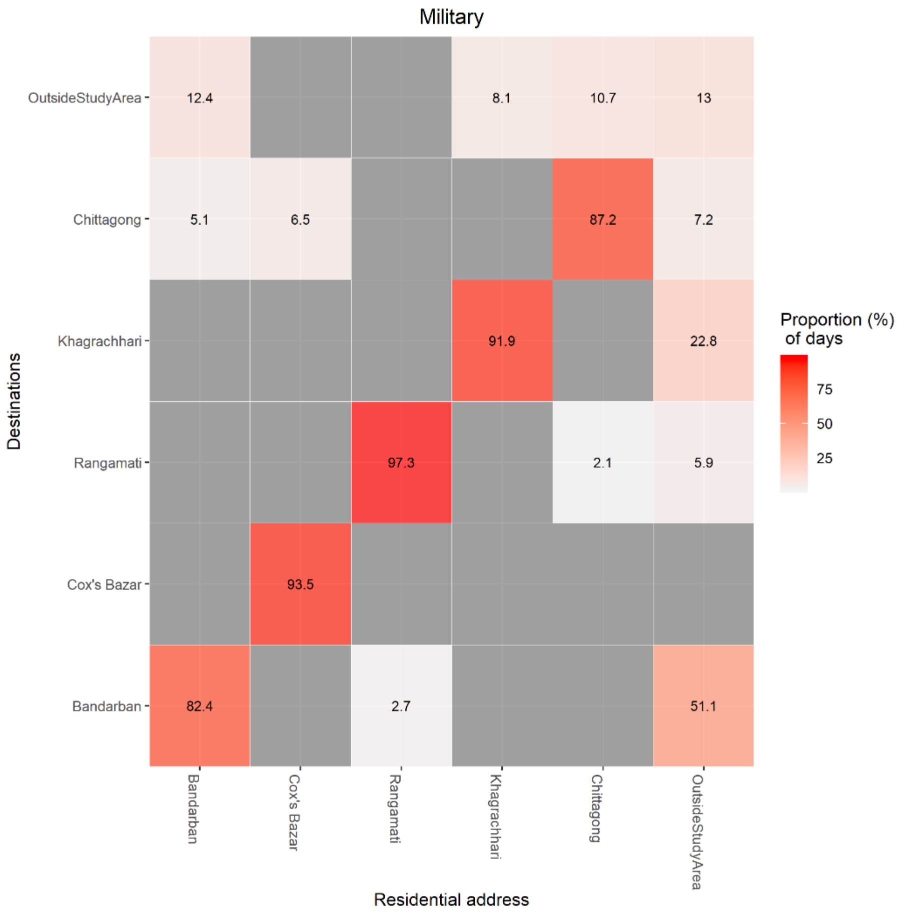

Figure S17 (a): Proportion of travel by days away from residence of military (n= 2928 days) at district level.

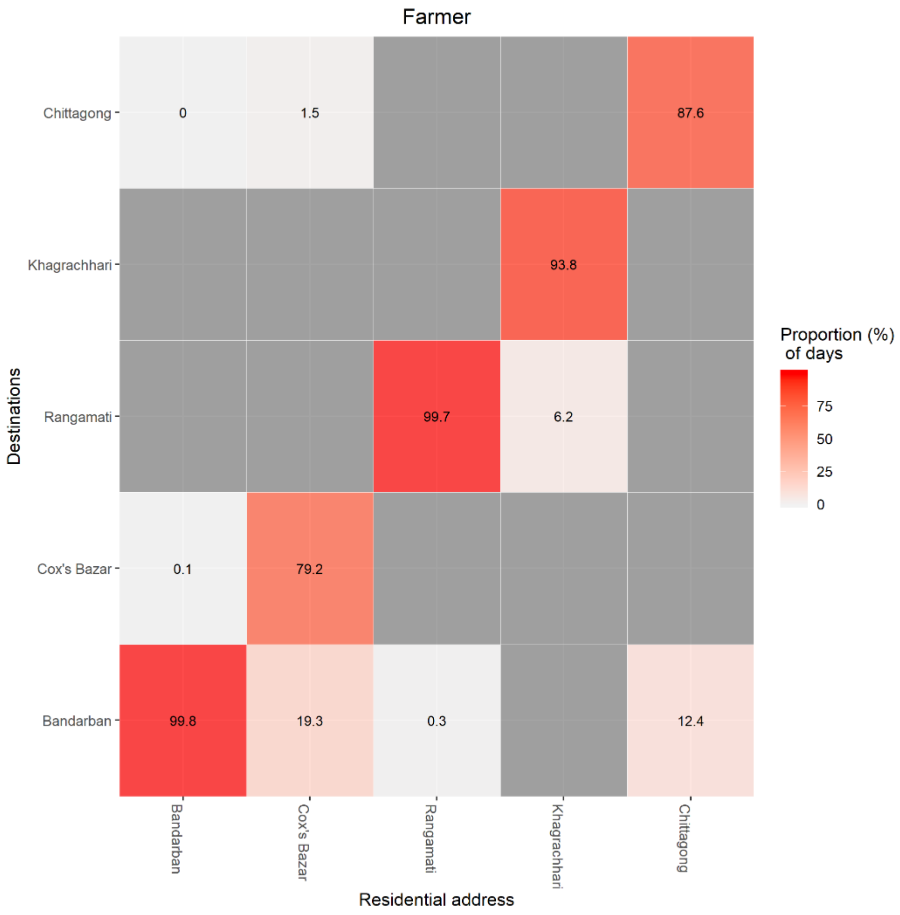

Figure S17 (b): Proportion of travel by days away from residence of farmers (n = 27409 days) at district level.

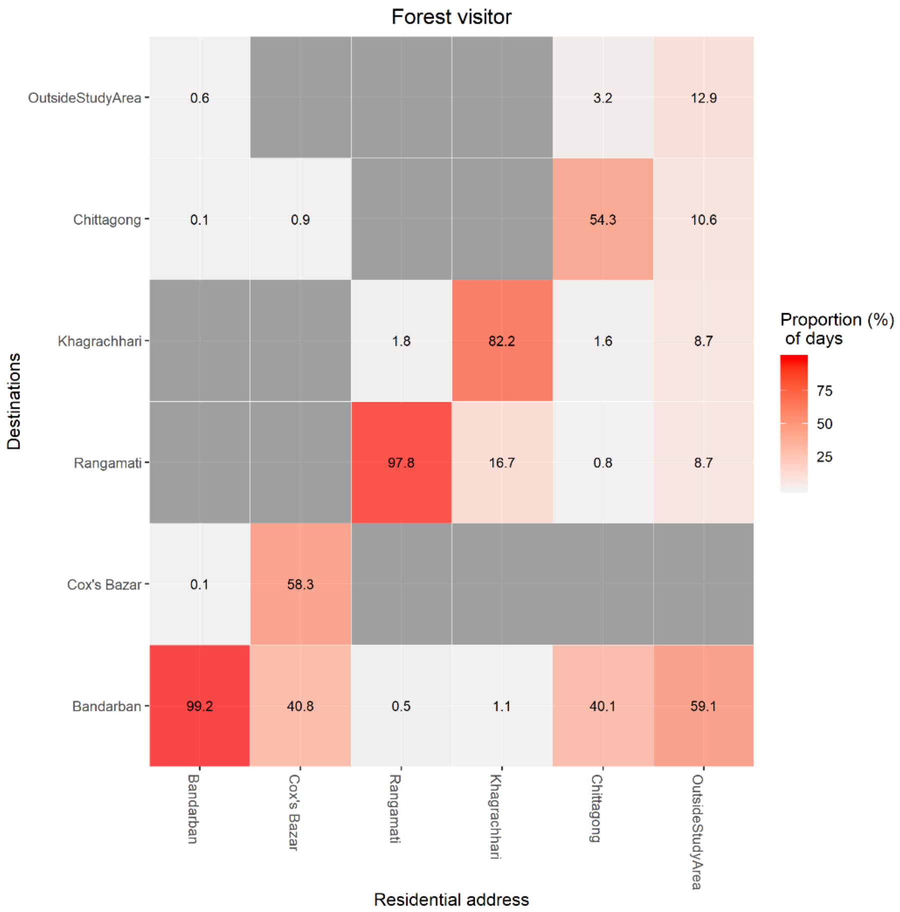

Figure S17 (c): Proportion of travel by days away from residence of forest visitors (n = 25449 days) at district level.

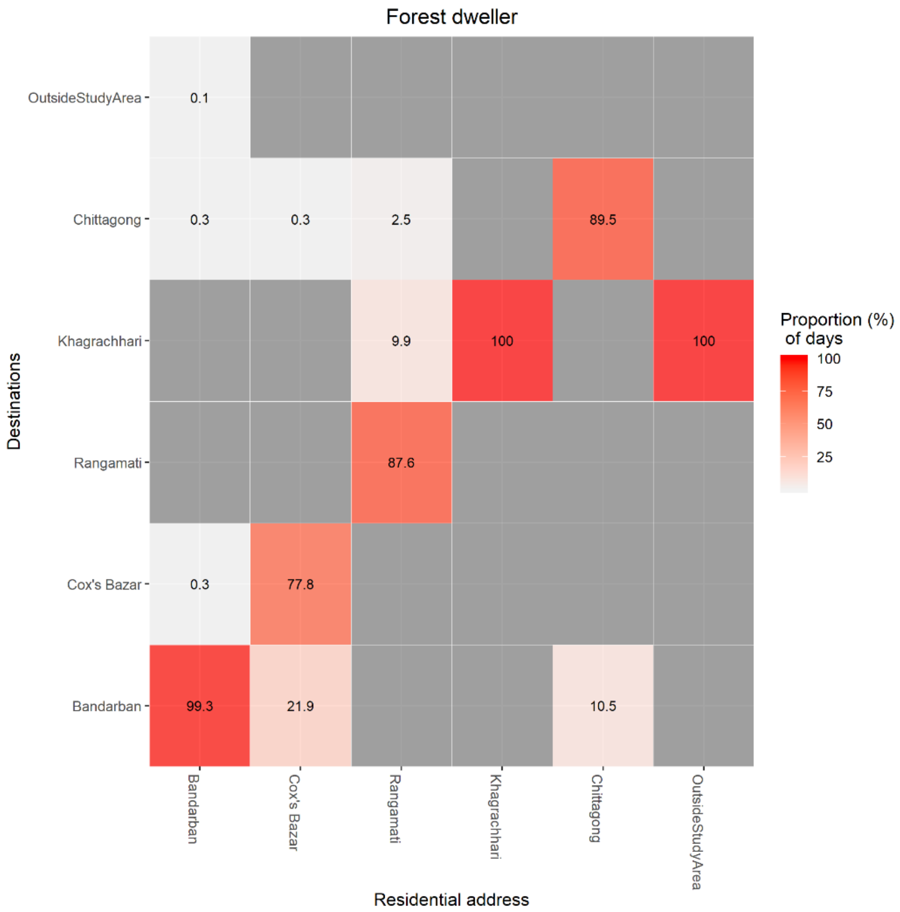

Figure S17 (d): Proportion of travel by days away from residence of forest dwellers (n = 20139 days away) at district level

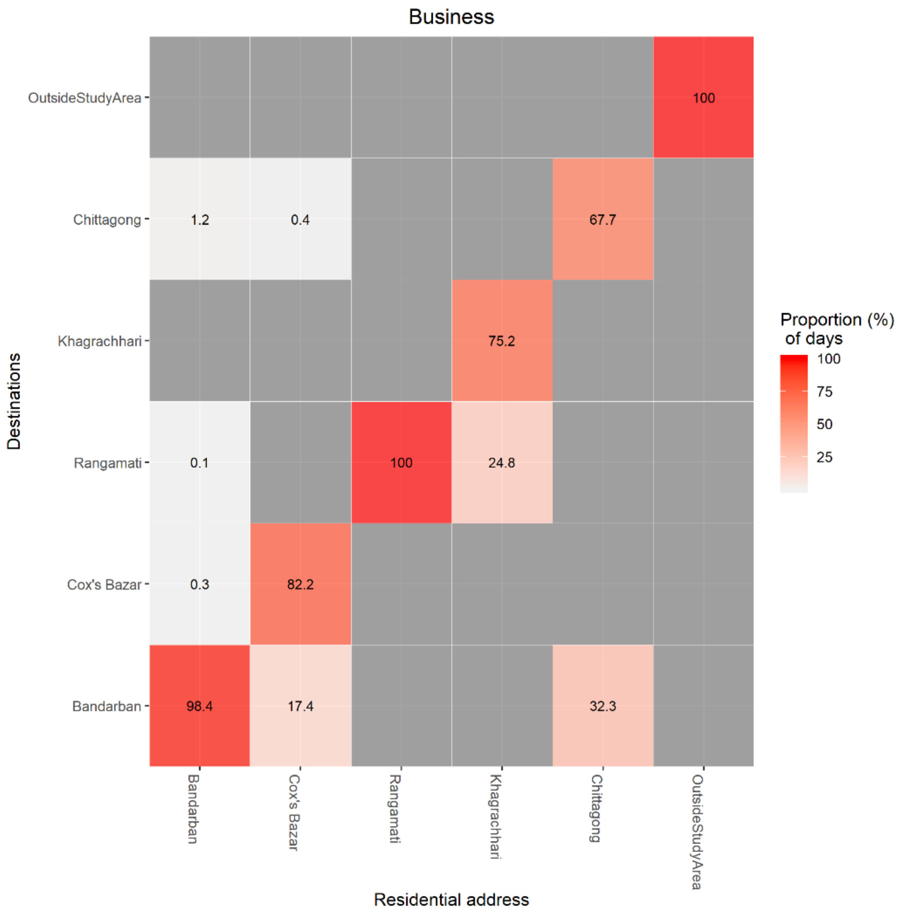

Figure S17 (e): Proportion of travel by days away from residence of business profession (n = 2955 days) at district level.

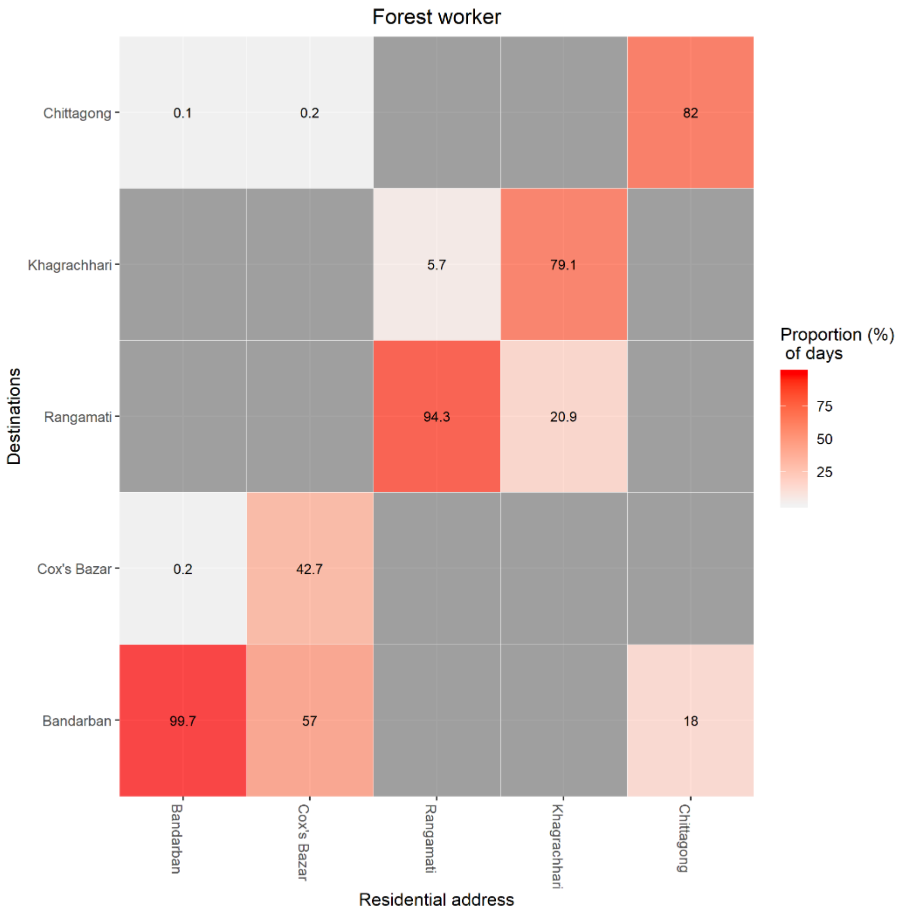

Figure S17 (f): Proportion of travel by days away from residence of workers reporting forest related occupations such as jhum cultivation, plantation or non-specific forest work (n = 21311) at district level

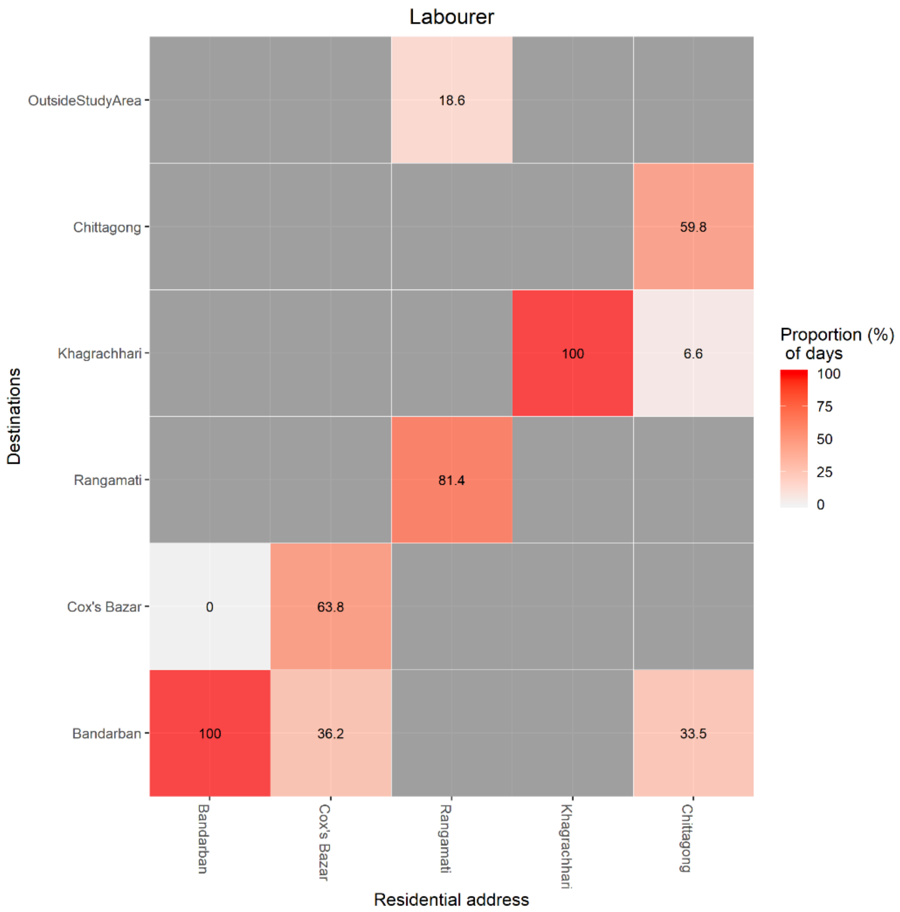

Figure S17 (g): Proportion of travel by days away from residence of labourers (n = 4829) at district level

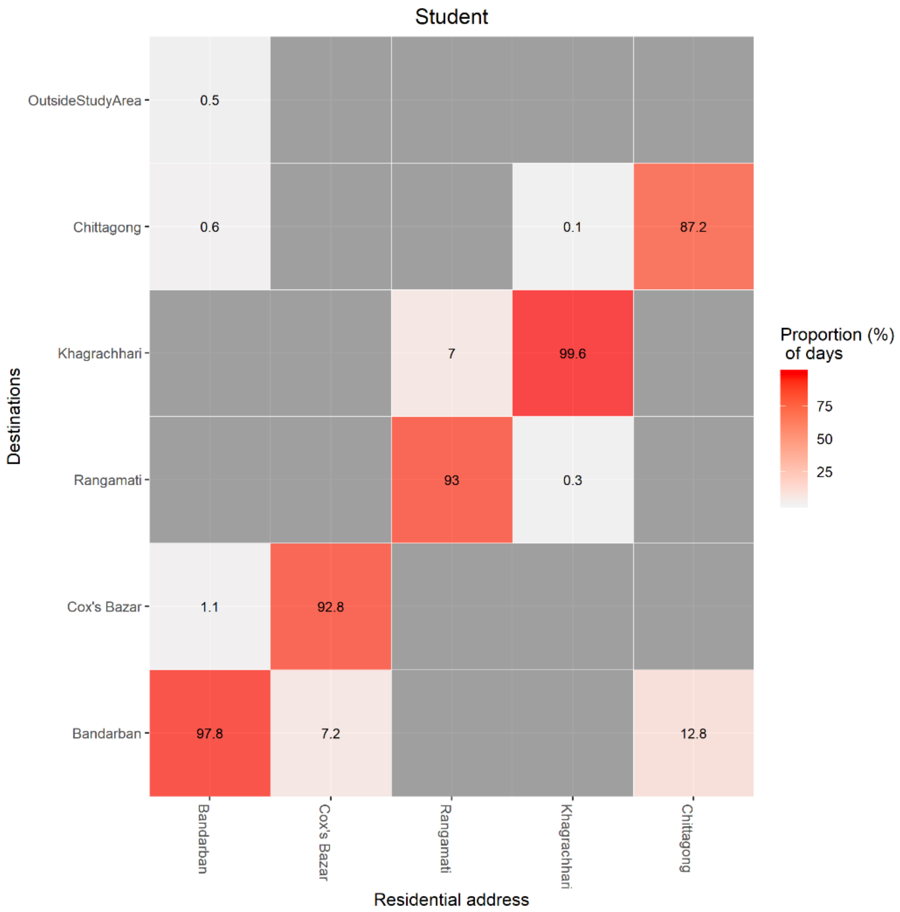

Figure S17 (h): Proportion of travel by days away from residence of students (n = 20207) at district level

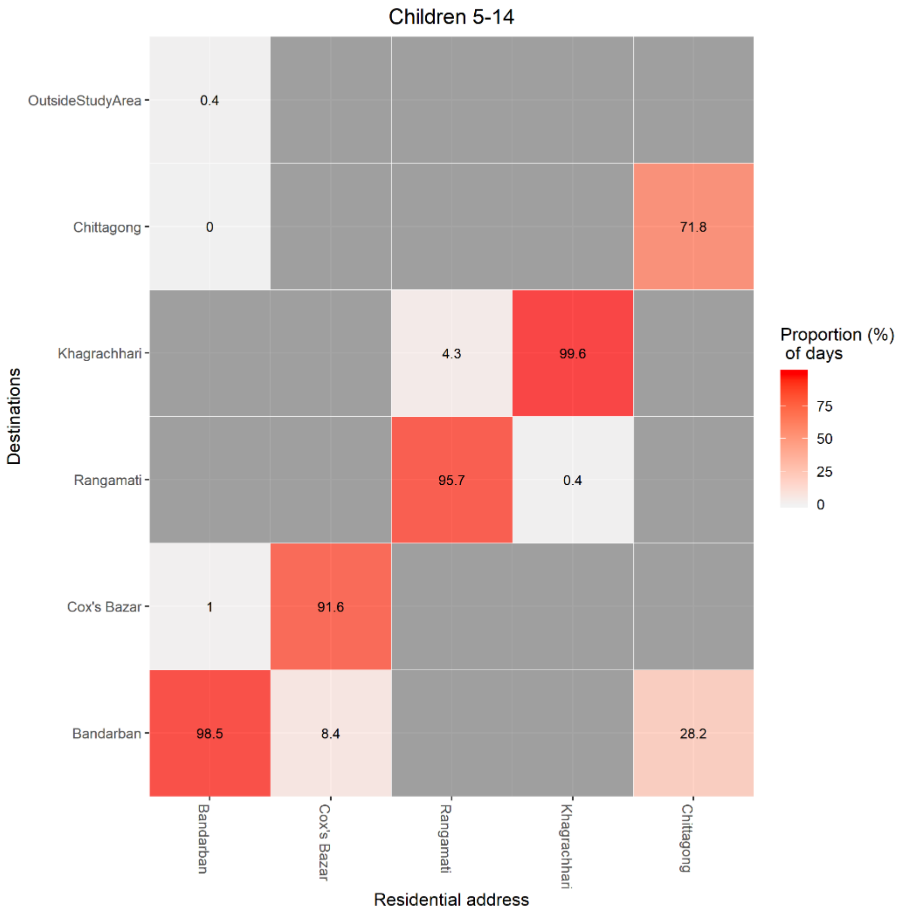

Figure S17 (i): Proportion of travel by days away from residence children aged 5-14 years (n = 21133) at district level

Travel and forest visits

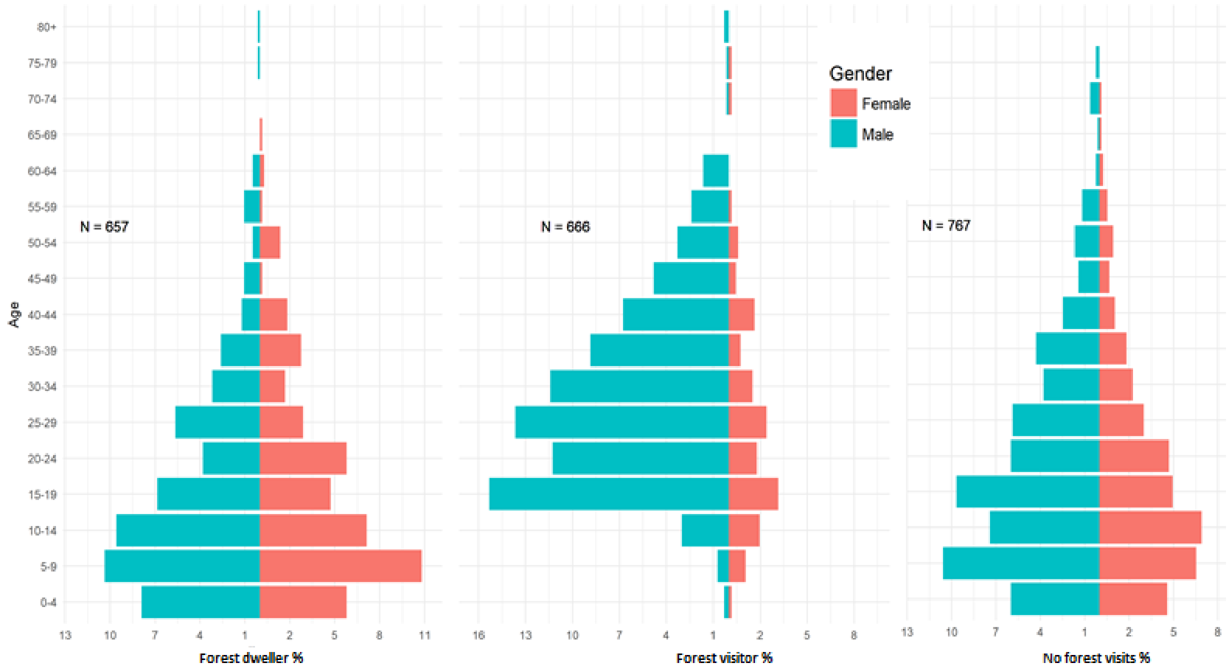

Figure S18: Forest residence and visit status by age and gender. Males aged 25-49 years were over-represented in the forest travel group, compared to people who did not visit the forest.

Both forest visitors and people not visiting the forest travelled further than people living in the forest, p < 0.001 (Figure S11).

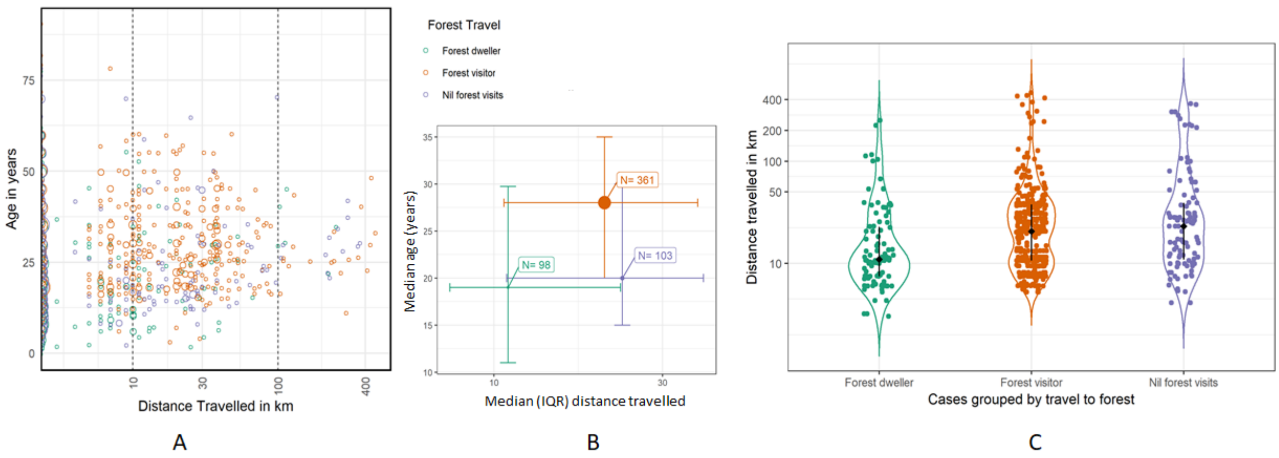

. Figure S19: (a) Distance (km) travelled by enrolled cases by forest status and age, (b) summarised by median and interquartile range (c) violin plot by forest status.

Table S14: Number of malaria cases by reported travel to forest and distance

	Number of cases		Days	Nights	Distance travelled by cases by km			
	Enrolled cases	Number of cases travelled (% of enrolled)	Number of days travelled	Number of nights away	25th percentile	50 percentile (median)	75th percentile	95th percentile
Forest dweller	657	427 (65%)	20,139	561	7	11	23	101
Forest visitor	666	666 (100%)	42,095	9,505	11	21	38	105
Nil travel to forest	767	538 (70%)	25,449	751	11	23	39	256

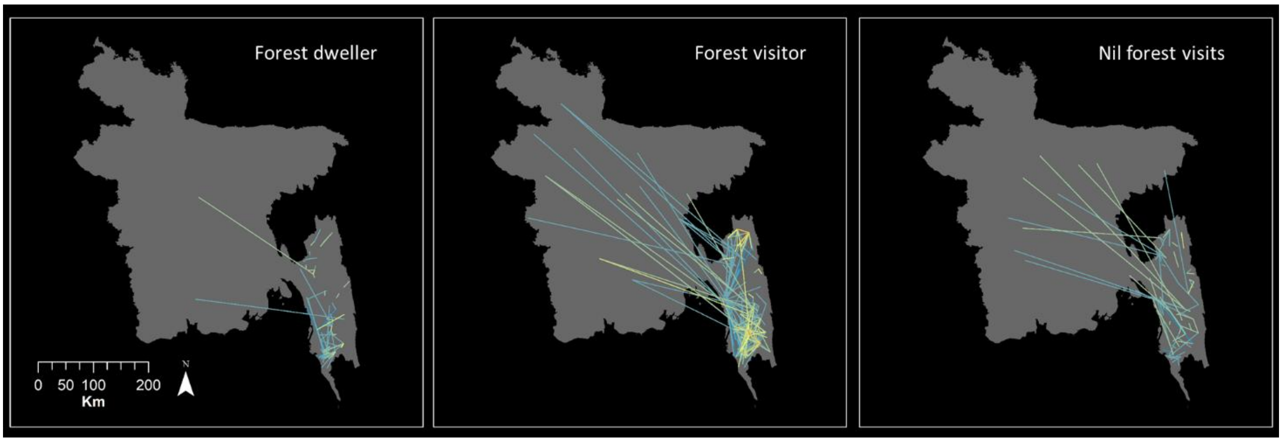

Figure S20: Overall days of travel over two months by forest reporting status.

Forest dwellers had proportionally more women (46%), then patients who did not visit the forest (38%), and the group visiting forest (16%, p<0.0001). Similarly, they were more children than adults in the forest dwellers group (54% children), with only 9% being children amongst the group visiting forest (p<0.0001).

Table S15: Number of malaria cases by reported forest status, gender and age

	Gender		
	Forest dweller	Forest visitor	Nil forest visits
Female	299 (46%)	106 (16%)	294 (38%)
Male	358 (54%)	560 (84%)	473 (62%)
	Age		
Age 0-15	353 (54%)	62 (9%)	346 (45%)
Age 15+	304 (46%)	604 (91%)	421 (55%)

Reasons for travelling to the forest

The geographic distribution of travel origins and destinations varied by reason cited for travel to forest. For example, plantation workers travelled mostly in the southern part of Chittagong division in Bandarban district (74% of travel days) mainly within Alikadam, Lama and Sarai unions. A smaller group of plantation workers (21%) travelled from Ramu, Cox's Bazar district to Naikhongchhari, Bandarban district. People who cut wood again travelled within the southern part of Chittagong division from Cox's Bazar district to Bandarban district (50% of travel days) followed by travel within Bandarban district (35%). For jhum cultivation, cases were distributed in both the north and the south of the Chittagong Hill Tracts with 45% travelling within Bandarban, followed by 20% in Rangamati and 15% in Khagrachhari districts.

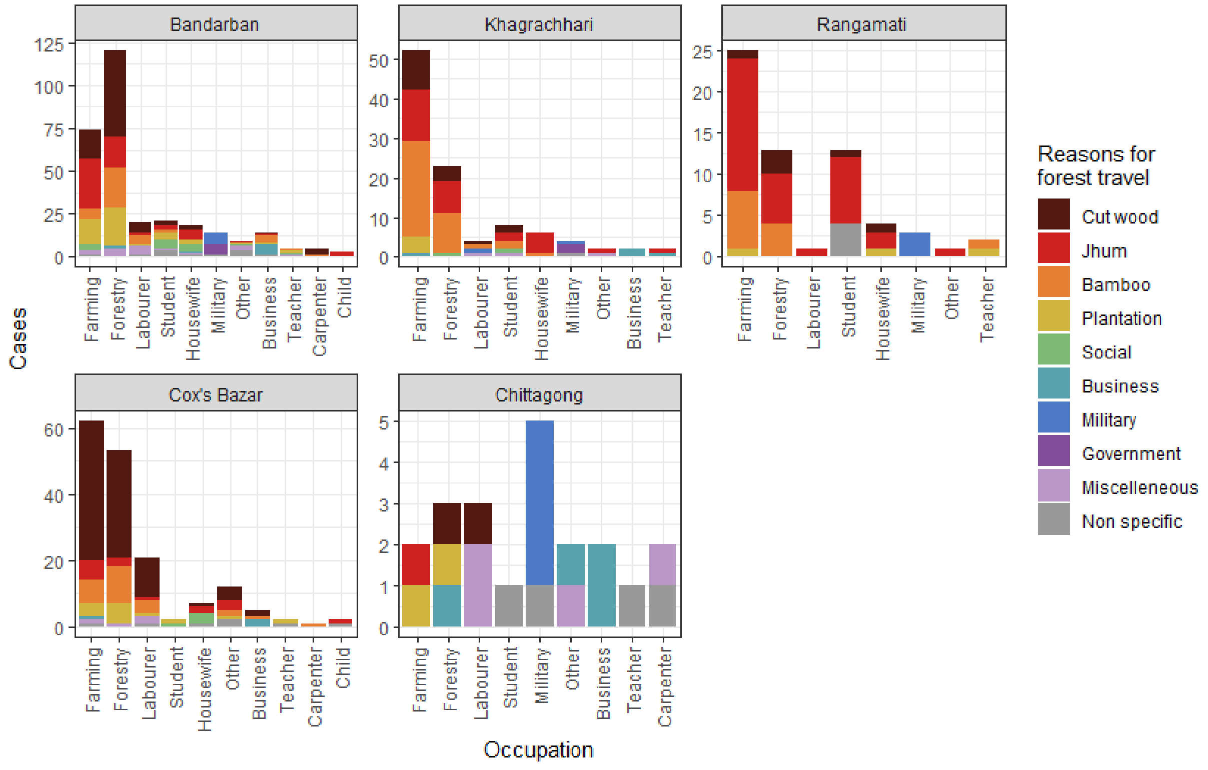

Figure S21: Reasons for travel to the forest by occupation and residential district

29 cases (4%) gave "travel" or a non-specific reason for travel to forest. Miscellaneous reasons included the following: animal keeping (6 people), work (6 people of which occupations were listed as labourer, teacher and government job), construction (4), labourer (3), fishing (2), collect

bananas (2), burn coal (1 person), carpenter (1), driver (1), health reasons (1), hunting (1), and religious reasons (1).

Reasons for non-work travel

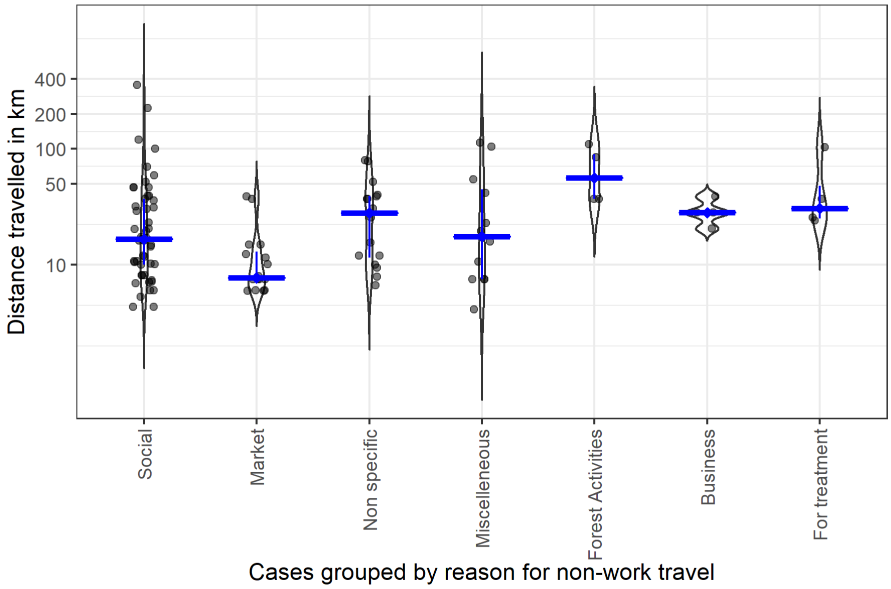

Figure S22: Distance travelled (km) and reasons reported for non-work travel (note 31 cases who reported non-work travel provided a non-specific reason for travel).

The number of cases who reported regular travel for purposes other than work or travel to the forest was 316 or 15% of the cases enrolled in the study with most patients engaged in travel for social purposes or trade (e.g. market). 31 cases (10%) gave “travel” or a non-specific reason for non-work travel. Miscellaneous reasons included the following: personal work (3 people), military (3), looking for a job (2), looking for information (2), religious reason (2), election (2 people), attending tutoring (1 person), driving (1), farming (1), for training (1), playing (1), poultry farm (1), seaside (1), supply wood (1), and to bring fertilizer (1).

Forest travel by percentage forest cover

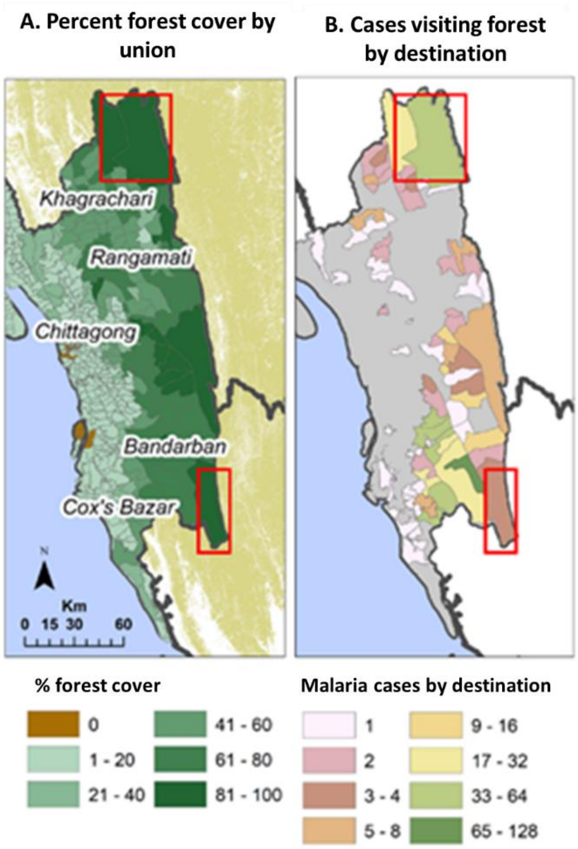

Figure S23: (a) percentage forest cover by union; (b) cases visiting the forest by destination union

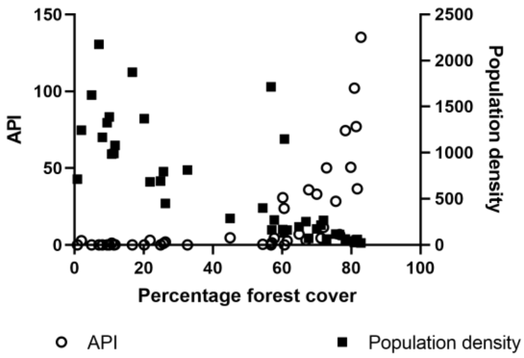

Figure S24: Graph showing percentage forest cover on x-axis, API at sub-district level for Chittagong division on x-axis (circles) and population density at sub-district level (squares). The population density is lower in higher percentage forest cover areas with higher API.
